# High-affinity PQQ import is widespread in Gram-negative bacteria

**DOI:** 10.1126/sciadv.adr2753

**Published:** 2025-05-30

**Authors:** Fabian Munder, Marcos Voutsinos, Klaus Hantke, Hari Venugopal, Rhys Grinter

**Affiliations:** ^1^ Department of Microbiology, Monash Biomedicine Discovery Institute, Monash University, Clayton, Australia.; ^2^ School of the Environmental Sciences, The University of Queensland, Brisbane, Queensland 4072, Australia.; ^3^ Faculty of Science, University of Tübingen, Tübingen, Germany.; ^4^ Ramaciotti Centre for Cryo-Electron Microscopy, Monash University, Clayton, Australia.; ^5^ Department of Biochemistry and Pharmacology, Bio21 Molecular Science and Biotechnology Institute, The University of Melbourne, Parkville, Victoria, Australia.; ^6^ Centre for Cryo-Electron Microscopy of Membrane Proteins, Monash Institute of Pharmaceutical Sciences, Parkville, Victoria 3052, Australia.

## Abstract

Pyrroloquinoline quinone (PQQ) is a soluble redox cofactor used by diverse bacteria. Many Gram-negative bacteria that encode PQQ-dependent enzymes do not produce it and instead obtain it from the environment. To achieve this, *Escherichia coli* uses the TonB-dependent transporter PqqU as a high-affinity PQQ importer. Here, we show that PqqU binds PQQ with high affinity and determine the high-resolution structure of the PqqU-PQQ complex, revealing that PqqU undergoes conformational changes in PQQ binding to capture the cofactor in an internal cavity. We show that these conformational changes preclude the binding of a bacteriophage, which targets PqqU as a cell surface receptor. Guided by the PqqU-PQQ structure, we identify amino acids essential for PQQ import and leverage this information to map the presence of PqqU across Gram-negative bacteria. This reveals that PqqU is encoded by Gram-negative bacteria from at least 22 phyla occupying diverse habitats, indicating that PQQ is an important cofactor for bacteria that adopt diverse lifestyles and metabolic strategies.

## Teaser

Diverse bacteria use the high-affinity membrane transport protein PqqU to scavenge the nutrient PQQ from the environment.

## INTRODUCTION

Pyrroloquinoline quinone (PQQ) is a soluble quinone redox cofactor used by a diverse family of calcium- and lanthanide-dependent dehydrogenases referred to as quinoproteins (*1*–*3*). Quinoproteins have been shown to oxidize substrates including sugars, alcohols, and aldehydes, and given the diversity of the family, it is likely that other substrate specificities exist (*4*–*9*). Quinoproteins are important metabolic enzymes in many environments, with PQQ-dependent methanol dehydrogenases the most abundant proteins found in some soil proteomes (*10*). These enzymes are predominantly found in Gram-negative bacteria, where they are localized to the periplasm and are sometimes membrane associated (*1*). The periplasmic localization of quinoproteins allows for the oxidation of catabolic substrates without their import into the cytoplasm, with the resulting electrons delivered to the respiratory chain via the reduction of C-type cytochromes, membrane-bound respiratory quinones, or azurin (*1*, *9*, *11*, *12*).

Endogenous PQQ is synthesized in the cytoplasm before it is transported to the periplasm where it is bound and used by quinoproteins (*13*–*15*). However, the molecular mass of PQQ (330 Da) is below the diffusion limit of the Gram-negative outer membrane, and the unbound cofactor can be lost from the cell via diffusion (*16*, *17*). As a result, many PQQ-producing bacteria secrete PQQ under conditions where its production is induced (*18*, *19*). This secretion makes PQQ a common good of microbial communities, a fact that is exploited by bacteria like *Escherichia coli*, which use PQQ-dependent enzymes but lack the biosynthetic enzymes for PQQ production (*20*).

The biosynthetic pathway for PQQ is complex, involving the production of the precursor peptide (PqqA), which is sequentially modified by four additional enzymes and a chaperone (PqqB to PqqF) to generate the cofactor (*13*). Considering the complex and energy-intensive nature of PQQ biosynthesis, the loss of PQQ from the cell by diffusion is energetically unfavorable, and as such, producing bacteria use systems to prevent the loss of PQQ from the cell or to recapture PQQ from the environment (*16*, *17*). An example of this is the periplasmic PQQ binding protein PqqT recently identified in the Alphaproteobacterium *Methylobacterium extorquens* AM1. PqqT binds PQQ with a high affinity (*K*_d_ = 50 nM), helping to prevent the loss of free PQQ from the cell and facilitate its capture from the environment (*17*).

PQQ can cross the outer membrane and enter the bacterial periplasm by diffusion. However, this process is inefficient, and unless the external concentration of the cofactor is high, it is unlikely to satisfy cellular requirements for the cofactor. Consistent with this, it was recently demonstrated that in *E. coli*, the TonB-dependent transporter (TBDT) PqqU (formerly YncD) enables the utilization of extracellular PQQ at concentrations as low as 1 nM, allowing phosphotransferase system–deficient cells to grow using the PQQ-dependent enzyme Gcd to oxidize glucose as the sole carbon/energy source (*16*, *21*). PqqU was not required for the growth of these cells at higher PQQ concentrations (100 to 3000 nM), indicating that PqqU is used for capturing the cofactor from the environment when concentrations are low. This provides strong evidence that PqqU is a high-affinity transporter of PQQ across the Gram-negative outer membrane (*16*). In addition to its role in PQQ import, PqqU acts as the cell surface receptor for several *E. coli*–targeting bacteriophages (*22*). Meanwhile, putative PqqU binding proteins have been identified in these phages based on the genome structure. The molecular basis for phage binding to PqqU remains unknown (*22*). PQQ blocks infection by the PqqU-targeting phage IsaakIselin, suggesting an interplay between the substrate and phage binding mechanisms (*16*).

In this study, we show that, consistent with its requirement for PqqU utilization at low concentrations, PqqU binds PQQ with a high affinity (*K*_d_ in the low to subnanomolar range). We determine the cryo–electron microscopy (cryo-EM) structure of the PqqU-PQQ complex at 1.99 Å, showing that extracellular loops 7 and 8 of PqqU undergo conformational changes to fully enclose PQQ in an internal binding pocket. Using structural modeling, we show that PQQ-induced conformational changes block the binding of a PqqU-targeting bacteriophage. We use the PqqU structure to generate mutants that identify key PQQ binding residues and perform phylogenetic analysis and structural modeling to map the presence of PqqU across Gram-negative bacteria. We find that PqqU is present in at least 22 phyla from diverse environments, and it is widespread in Gammaproteobacteria, Bacteroidota, and Gemmatimonadetes. Only 23% of bacteria encoding PqqU also encode the biosynthetic machinery for PQQ biosynthesis, indicating that PQQ scavenging is an efficient strategy for diverse bacteria. PqqU producers often, but not always, encode putative quinoproteins, indicating that there may be additional uses for the cofactor outside this enzyme family. These findings indicate that PQQ is an important nutrient for bacteria adopting diverse metabolic strategies and that high-affinity PQQ import is a beneficial trait in many environments.

## RESULTS

### PqqU binds PQQ with a high affinity

**Fig. 1.**
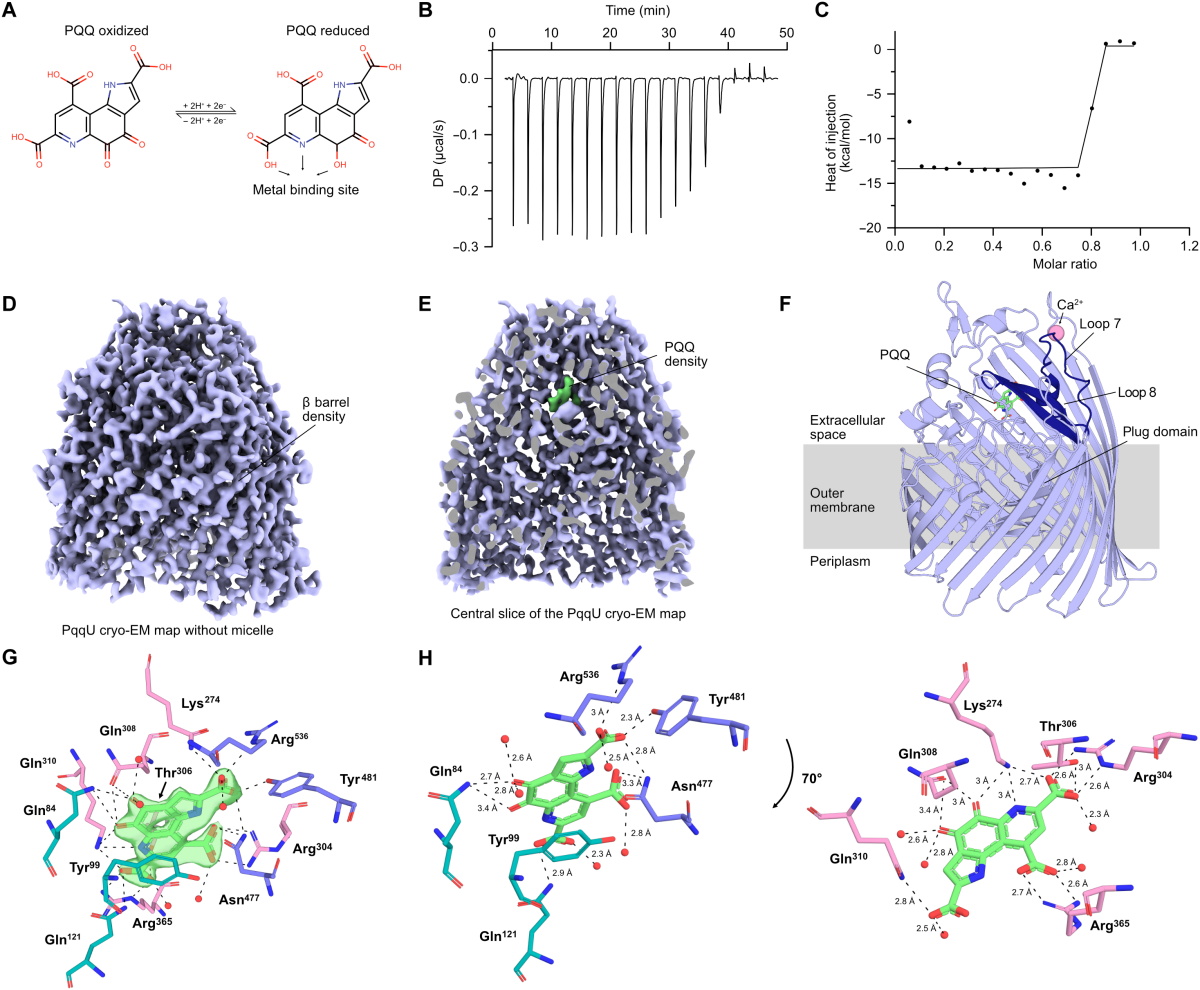
PqqU binds PQQ with a high affinity by enclosing it in an internal binding cavity. (**A**) PQQ molecule visualized via a 2D stick model. PQQ changes from the oxidized form to the reduced form upon the two-electron transfer to both hydroxyl groups. The first aromatic ring contains nitrogen that, together with two carbonyl groups, coordinates a metal ion (Ca^2+^ or lanthanide) in PQQ-dependent enzymes (quinoproteins). (**B**) Representative thermogram of 100 μM PQQ titrated into 20 μM PqqU over ~50 min in 19 injections of 2 μl. The first injection (not included in the graph) is 0.4 μl. DP, differential power. (**C**) Negative peaks of injections integrated and plotted against the PqqU-PQQ molar ratio, excluding the first injection. (**D**) The PqqU coulomb potential map reveals distinct structures of PqqU such as the characteristic β barrel made of 22 individual β strands. The LMNG micelle has been removed for clarity. (**E**) Vertical cut of the PqqU coulomb potential map to reveal the density of a single PQQ molecule (green). (**F**) Cryo-EM structure of PqqU with bound PQQ (green) in the closed conformational state at a global resolution of 1.99 Å. Extracellular loops 7 and 8 of PqqU undergo a conformational change that encloses the substrate binding pocket (dark blue). Visualized as a side view with a slight tilt to expose the PQQ molecule. (**G**) PQQ (green) with map density surrounded by the 12 binding site residues colored by location within PqqU (β barrel, pink; loops 7 and 8, blue; plug domain, green). Interaction distances between PQQ and residues/waters visualized by dotted lines. (**H**) Rotational view of PQQ with the 11 binding site residues and bond lengths. Split into two parts for visual clarity based on location within the binding pocket.

Previous work showed that PqqU is required for high-affinity uptake of the redox cofactor PQQ (Fig. 1A) (*16*). However, this study did not provide direct evidence for interaction between PqqU and PQQ, which would be required for import. To verify that this interaction is occurring and to gain insight into its affinity and binding kinetics, we performed isothermal titration calorimetry (ITC), with PQQ injected into purified PqqU in the detergent lauryl maltose neopentyl glycol (LMNG) (fig. S1). Free LMNG micelles were depleted from the PqqU sample in these experiments to ensure buffer matching. PQQ in these experiments and throughout this study was stored and handled under ambient conditions. The standard redox potential of PQQ is −0.125 V, meaning that it was maintained in an oxidized state because of the presence of O_2_ in the atmosphere (*23*, *24*). The ITC thermogram from this experiment shows negative saturable heats of binding, indicative of an exothermic interaction between PqqU and PQQ (Fig. 1B), with minimal heats observed for the buffer into PqqU control (fig. S2). Analysis of these data revealed a molar binding ratio of 0.74 PQQ molecules per PqqU, which likely indicates a 1:1 stoichiometry for this interaction, with not all PqqU molecules in a binding-competent state after dialysis in detergent-free buffer. A Gibbs free energy (Δ*G*) of −16.4 kcal/mol was calculated for PqqU-PQQ binding, which is largely driven by a favorable enthalpy of binding of −14.4 kcal/mol. This is consistent with the negatively charged nature of PQQ and the positively charged nature of the putative substrate binding site of PqqU (*1*, *21*). A binding curve could not be accurately fitted to the integrated heats from the ITC titration because of a lack of data across the inflection point, indicating that the interaction between PqqU and PQQ is of high affinity, likely with a *K*_d_ in the low to subnanomolar range (Fig. 1C and table S1). This is consistent with the biological role of PqqU as a high-affinity PQQ transporter (*16*).

### High-resolution cryo-EM structure of the PqqU-PQQ complex

**Fig. 2.**
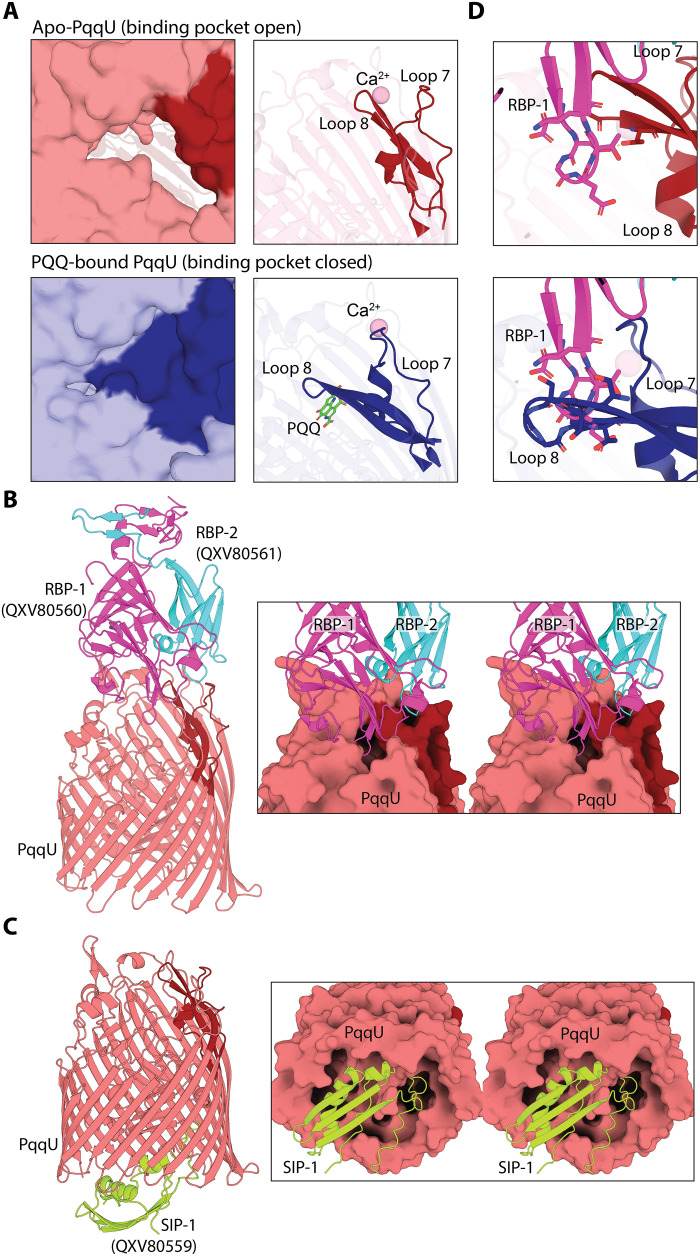
PQQ capture by PqqU prevents binding of the PqqU-targeting phage. (**A**) Close-up comparison of the surfaces of PqqU in open apo (red) and closed PQQ-bound (blue) states (left). In the closed PQQ-bound state, PQQ is obscured by loop 8. Close-up cartoon of PqqU in the closed conformational state (blue) compared to the open conformational state (red) visualizing the movement of loops 7 and 8 upon PQQ binding (right). (**B**) AlphaFold2 prediction of the extracellular side of PqqU in complex with two predicted IsaakIselin phage receptor binding proteins RBP-1 (pink) and RBP-2 (cyan). Cartoon view of the predicted complex (left) and zoomed cross-eye stereo view of the interface of the complex shown with RBP-1 and RBP-2 as cartoon and PqqU as a molecular surface (right). (**C**) AlphaFold2 prediction of the periplasmic side of PqqU in complex with the putative superinfection blocking protein SIP-1 (yellow), shown as in (B). (**D**) The structure prediction indicates that loops 7 and 8 are open during RBP-1 and RBP-2 binding, with clashes observed with loop 8 in the closed state.

To determine the structural basis for the high-affinity binding of PQQ by PqqU, we used cryo-EM to resolve the structure of the PqqU-PQQ complex. PqqU in LMNG in micelle-depleted buffer was mixed with a 20-fold excess of PQQ before grid preparation and imaging on a Titan Krios microscope. Image processing and three-dimensional (3D) reconstruction yielded high-quality maps of PqqU with a nominal resolution of 1.99 Å (Fig. 1, D and E, fig. S3, and table S2). These maps allowed us to model the majority of the polypeptide chain of PqqU (amino acids 39 to 699 of 700 total), confirming that PqqU forms a monomeric 22-stranded β barrel, with a transmembrane region enclosed in an LMNG micelle (Fig. 1F, fig. S4, and movie S1). This high-resolution structure of PqqU, while globally similar to the PQQ-free structure previously solved by x-ray crystallography [YncD; Protein Data Bank (PDB) ID: 6V81; root mean square deviation, 0.957; 4030 of 5147 atoms], reveals conformational changes in extracellular loops 7 and 8 and a general compaction of the transporter (Fig. 2A and movie S3). We observed unambiguous map density corresponding to a single PQQ molecule within the extracellular region of PqqU that was previously identified as a potential substrate binding pocket (Fig. 1E) (*21*). No density is observed for a metal ion in complex with PQQ, indicating that PqqU does not cotransport the compound with a metal cofactor despite commonly forming a catalytic complex with Ca^2+^ or La^3+^ in PQQ-dependent enzymes (*1*, *2*). However, PqqU extracellular loop 6 coordinates a metal ion, which we modeled as a Ca^2+^ ion based on the analysis of the structure and density maps using the Check My Metal Server (*25*). This putative Ca^2+^ ion may play a role in an earlier phase of PQQ binding (Fig. 1F). A total of 12 amino acids from PqqU interacts with PQQ and originates from extracellular loops 7 and 8, the N-terminal plug domain, and the β barrel (Fig. 1, G and H). In addition, the high resolution of the cryo-EM data allowed us to resolve five ordered water molecules directly coordinating PQQ. Three arginine residues R304, R365, and R536 interact with the carboxylic groups of PQQ, contributing to the binding of PQQ by charge attraction. A plug domain–located glutamine Q84 interacts with the two carbonyl groups of the central aromatic ring of PQQ, which may be important during the release of PQQ, while the plug domain tyrosine Y99 forms a π-π stacking interaction with the aromatic ring of PQQ. Q84, Y99, and Q121 may tether PQQ to the plug domain, facilitating its entry into the periplasm when the transporter is energized by TonB, which leads to the displacement of the plug domain and formation of the transmembrane transport pore (*26*–*30*).

### Conformational changes in PqqU loops remodel the transporter to capture PQQ

In its unbound state, the substrate binding pocket of PqqU forms a deep cleft, which is open to the extracellular environment. In contrast, in the substrate-bound structure, PQQ is entirely enclosed within the substrate binding pocket and is not accessible to the external environment (Fig. 2A). This change in accessibility of the PqqU substrate binding pocket is largely mediated by major conformational changes in extracellular loops 7 and 8, which move inward to form a barrier between the PQQ binding pocket and the external environment (Fig. 2A and movie S2). This change in the conformation of loops 7 and 8 is accompanied by a general compaction of PqqU around PQQ, as well as changes in the orientation of binding pocket side chains to form the highly coordinated PqqU-PQQ complex (Fig. 1, G and H, and movie S3). Notably, Y99 and Q121 undergo a near-180° flip to interact with PQQ where Y99 forms the π-π interaction with the face to PQQ (movie S3). These conformational changes are triggered by the binding of PQQ to the open state of PqqU, with our substrate-bound structure representing an intermediate state in the transport pathway, before substrate import facilitated by energy provided by interaction with TonB (*26*–*28*, *30*). The closure of the substrate binding pocket may facilitate a double-gated import mechanism, suggested for other TBDTs, where the PqqU transporter channel is not simultaneously open toward the periplasm and extracellular space, preventing the inadvertent import of deleterious compounds (*28*, *29*, *31*, *32*).

Previous studies found a family of bacteriophages that infect *E. coli* by using PqqU as their cell surface receptor and identified that PQQ can block infection by one of these phages (*16*, *22*). A 1000-fold lower PQQ concentration is required to block infection in an *E. coli* Δ*tonB* strain, in which PqqU cannot be activated for import (*16*). Given the high affinity of the PqqU-PQQ interaction and the closure of the binding pocket upon substrate binding, PqqU in the Δ*tonB* strain likely remains in the closed PQQ-bound state indefinitely, suggesting that the closed state of PqqU identified by our structure is not competent for phage binding. To determine the structural basis for the observation, we used AlphaFold2 to predict the complex of PqqU and three putative receptor binding proteins encoded by the PqqU-targeting phage IsaakIselin (*22*, *33*, *34*). Notably, AlphaFold2 predicted that PqqU forms a complex with all three phage proteins with high confidence (Fig. 2, B and C, fig. S5, and data S1). Two of the phage proteins (RBP-1 = QXV80560; RBP-2 = QXV80561) dimerize to form a complex with the extracellular face of PqqU, together constituting the phage receptor binding domain (Fig. 2B). In the AlphaFold2 model, the phage receptor binding domain binds to PqqU in the open state (Fig. 2D). Many clashes occur between loops 7 and 8 of PqqU and the phage receptor if the closed PQQ-bound structure of PqqU is superimposed with this model. These clashes would likely preclude binding, explaining the observed resistance to the phage when PQQ is present (Fig. 2D) (*16*). These data indicate that the closed PQQ-bound state we observe in our structure is likely irreversible without energy input from TonB, which supports our proposed double-gated import mechanism for PQQ import through PqqU.

In contrast, the third protein (SIP-1 = QXV80559) forms a complex with the intracellular side of PqqU and likely constitutes a superinfection exclusion factor analogous to Llp from the T5 phage (Fig. 2C) (*35*). Once a bacterial cell is infected with a bacteriophage, superinfection exclusion factors function to prevent further phage infection, often by blocking binding at cell surface receptors (*35*). As such, SIP-1 is likely produced in the bacterial cytoplasm by cells infected with phage IsaakIselin before transport to the periplasm where it binds to PqqU, inducing a conformational change that blocks phage binding. Consistent with this, the N terminus of SIP-1 is predicted by SignalP-6.0 to encode a signal peptide (*36*). Foldseek analysis of the QXV80559 structural model reveals that it is structurally homologous to the C terminus of TonB, which binds TBDTs and provides the mechanical force required to drive substrate import (*26*, *37*). This indicates that the IsaakIselin phage has likely repurposed this domain of TonB to inactivate PqqU and block superinfection.

### Electrostatic and π-π interactions are critical for PQQ transport by PqqU

**Fig. 3.**
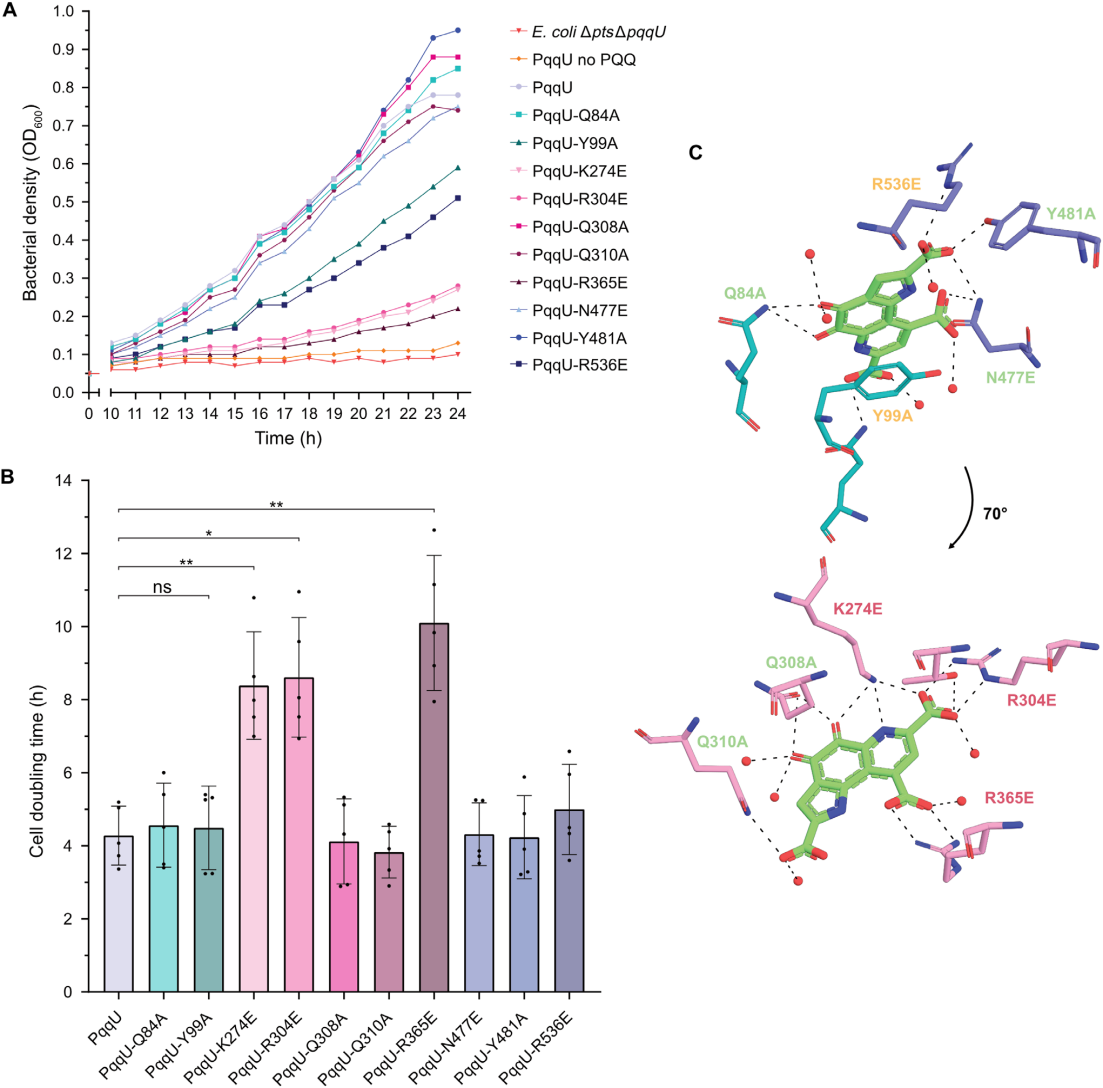
Binding site mutations of PqqU reduce the ability to perform Entner-Doudoroff pathway glycolysis. (**A**) Representative growth curve of *E. coli* Δ*pts*Δ*pqqU* cultures grown in M63 minimal media containing 0.1% glucose and supplemented with 10 nM PQQ, unless indicated. The cells were transformed with plasmids containing mutated binding site variants of *pqqU*. h, hours. (**B**) The doubling time of cells was individually calculated from the exponential growth phase of each culture (*n* = 5, biological replicates). Statistical differences between PqqU and mutated PqqU were determined by performing paired *t* tests (ns, not significant; *P < 0.05 and **P < 0.01). (**C**) Rotational view of PQQ with the 12 binding site residues of PqqU. The mutated residues and their effect on culture growth are colored on the basis of severity.

To test the relative importance of residues in the PQQ binding site for substrate import, we generated PqqU variants where these residues were mutated either to alanine or, in the case of K274, R304, R365, N477, and R536, to glutamate (Fig. 3). An inducible plasmid encoding these variants was transformed into a phosphotransferase system–deficient *E. coli* strain also lacking PqqU (*E. coli* Δ*pts*Δ*pqqU*). This strain requires the PQQ-dependent enzyme Gcd to grow when glucose is its sole energy and carbon source, which makes PqqU essential when external PQQ concentrations are low (<100 nM) (*16*). Under these conditions, after a pronounced lag phase (~10 hours), the cell growth of both wild-type and mutant PqqU-expressing cells was measured by optical density. In the absence of exogenous PQQ, *E. coli* Δ*pts*Δ*pqqU* complemented with wild-type PqqU exhibited minimal growth, whereas, in the presence of 10 nM PQQ, the strain grew to a final optical density (OD) of ~0.8, with a doubling rate of ~4 hours. The exponential growth rate of strains complemented with several PqqU binding site variants (Y481A, Q308A, Q84A, Q310A, and N447E) was comparable to that of the wild type, indicating that these residues are not critical for PQQ uptake by themselves. Conversely, a longer lag phase and slower growth were observed for strains containing PqqU variants K274E, R304E, and R365E despite comparable expression to wild-type PqqU (Fig. 3, A and B, and fig. S6). These residues are in the β barrel portion of the binding pocket and form salt-bridge interactions with PQQ, which indicates that they are likely crucial in capturing the substrate. While PqqU variants R536E and Y99A did not exhibit a notably slower growth rate during the exponential phase, these variants exhibited a longer lag phase, indicating that they are important for PQQ binding or transport (Fig. 3, A and B). Y99A is in the plug domain and forms direct π-π stacking with polycyclic PQQ, stabilizing the cofactor during binding and, possibly, import. These data indicate that while this interaction is important for the PqqU function, its substitution for alanine is not as detrimental as charge swap mutations of R304 and R365 that interact with PQQ carboxylic acid groups. In summary, this section identified key residues in the PqqU binding site for PQQ import, providing mechanistic insight into transporter function and providing a blueprint for identifying PQQ transporters in other bacteria.

### PqqU is present in diverse Gram-negative bacteria

**Fig. 4.**
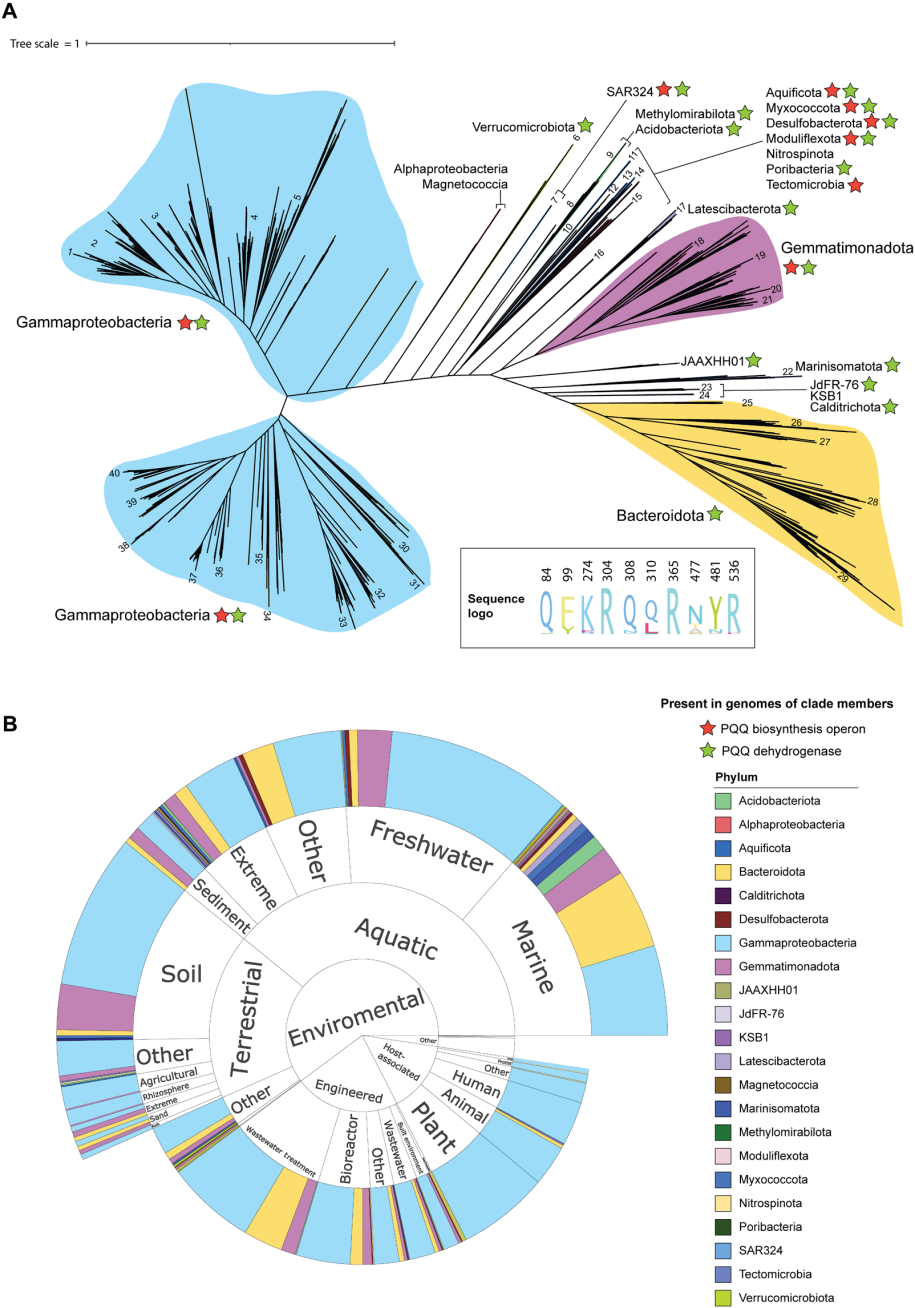
Phylogeny and origin of PqqU-containing bacteria. (**A**) Phylogenetic tree of bacteria encoding PqqU identified by our homology search of bacterial genomes. The tree was constructed on the basis of 16 ribosomal proteins. The classes of bacteria encoding the various clades of PqqU are shown, as well as the presence of genes encoding PQQ biosynthetic capacity or quinoproteins (PQQ-dependent dehydrogenases) in at least some of the members of that clade. Bacteria containing PqqU sequences used for structural modeling in complex with PQQ are numbered; see table S3 for sequence IDs. A sequence logo showing the conservation of PQQ-interacting residues across all identified PqqU homologs is shown as an inset; see fig. S8 for a full consensus logo and fig. S4 for a more detailed view of this tree. (**B**) Sunburst plot showing the environment of origin/isolation for bacteria encoding PqqU.

TBDTs import diverse substrates and are highly variable in the amino acid sequence and outer loop structure, even between transporters targeting the same substrate (*26*, *38*–*40*). As a result, it is difficult to definitively determine substrate specificity based on phylogenetic relationships and global sequence identity alone. To overcome this and identify PqqU in Gram-negative bacteria, we used the key conserved PQQ binding residues Y99, R304, and R365 as a molecular signature for PqqU to identify the presence of genes encoding the transporter in publicly available genomes and metagenome-assembled genomes (Fig. 3). As Y99 is responsible for π-π interactions with PQQ, we also accepted phenylalanine and tryptophan in this position, as they will also satisfy this interaction. While K274 and R536 are important for the PqqU function in *E. coli*, they are less conserved, so we did not use them to filter putative PQQ transporters (fig. S7). After genome dereplication, PqqU was identified in 1861 diverse Gram-negative bacterial isolate genomes and metagenome-assembled genomes, representing 22 phyla. In addition, we analyzed the genomes of these PqqU-producing isolates for the genes responsible for PQQ biosynthesis (PqqA to PqqF) and PQQ-dependent dehydrogenases to gain insight into the role of PqqU in these organisms (Fig. 4A, fig. S8, and data S2 and S3).

**Fig. 5.**
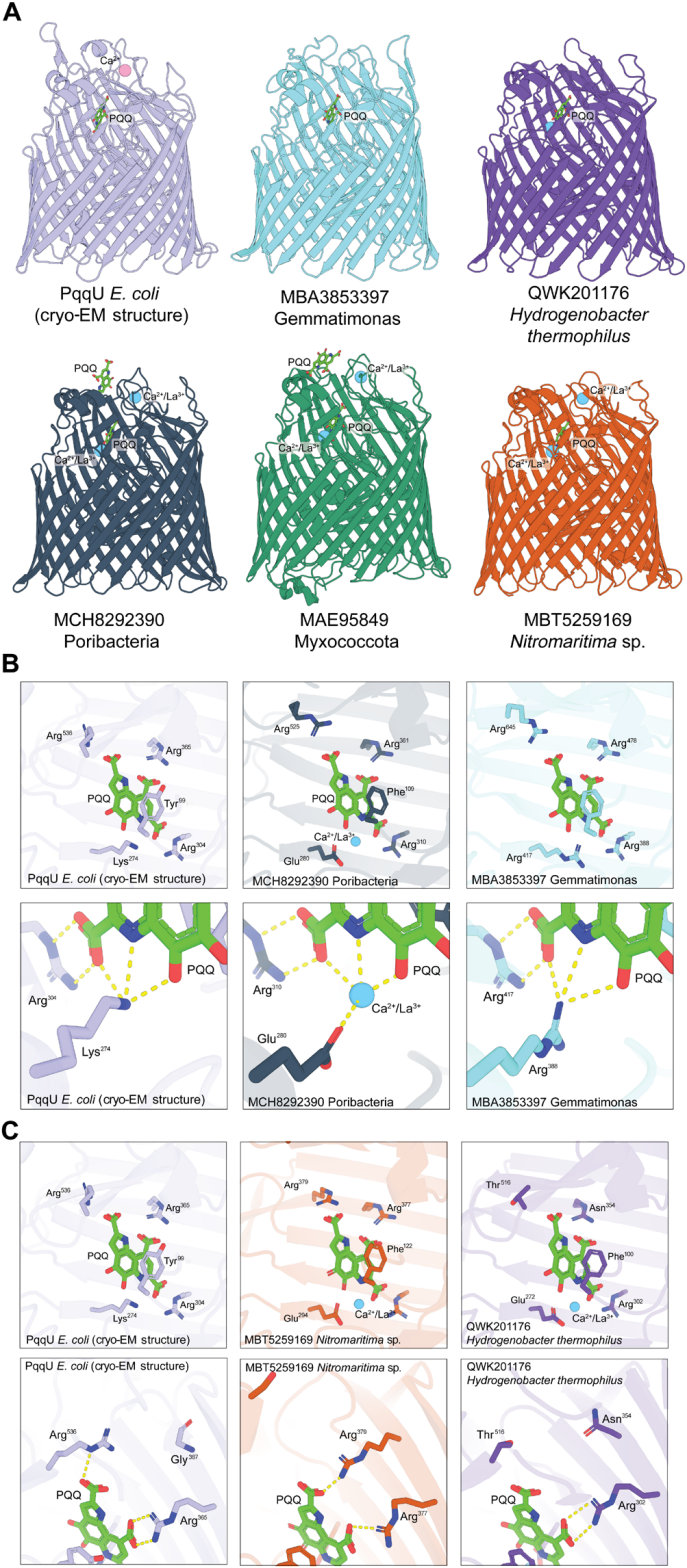
Chai-1 modeling indicates that diverse PqqU homologs bind PQQ. (**A**) Comparison of the experimental structure of the PqqU-PQQ complex from *E. coli* with a selection of Chai-1 models of diverse PqqU-PQQ complexes (see data S4 for coordinate files for all models). (**B**) Comparison of models with glutamate and arginine substitutions as the position equivalent to K274 in *E. coli* PqqU. When glutamate substitutes lysine, a predicted metal ion binding site is formed, coordinated by PQQ and the glutamate. (**C**) A comparison of models lacking the semiconserved R536 in *E. coli* PqqU shows that the interaction between this residue and PQQ either is compensated for by arginine at another position in PqqU or is absent.

To validate that the PqqU homologs identified in our phylogenetic analysis function as PQQ importers, we performed structural modeling on a subset of 42 of these proteins in complex with PQQ using the program Chai-1 (Fig. 5A and fig. S9) (*41*). The sequences used were representative of the diversity of the PqqU homologs we identified (Fig. 4A, table S3, and data S4). For all TBDTs, Chai-1 modeled PQQ in the substrate binding pocket of the transporter in an analogous binding mode to PQQ in our cryo-EM structure of the *E. coli* PqqU-PQQ complex (fig. S9). In some cases, when one PQQ molecule was specified, it was modeled in a semiconserved binding site of the surface of the transporter (fig. S10A). However, when two PQQ molecules were modeled, the second PQQ was positioned at the conserved binding site. This first binding site may represent a secondary substrate binding site, as observed for other TBDTs (fig. S10A) (*32*, *42*, *43*). The global predicted template modeling (pTM) confidence scores and predicted local distance difference test (pLDDT) scores for the transporter polypeptide were high, indicating that this modeling is reliable. In most cases, the pLDDT score for PQQ exceeded 80, indicating confidence in the modeling of this ligand (table S3 and data S4). Critically, the positioning of amino acids equivalent to Y99, R304, and R365 from *E. coli* PqqU was analogous to the cryo-EM structure, with these residues interacting with the same functional groups of PQQ (Fig. 5A and fig. S9). A considerable fraction of the models has glutamate substituting K274. This substitution is curious as it swaps the charge of this side chain from positive to negative and was found to inactivate *E. coli* PqqU in our phenotypic assays (Fig. 2).

As a catalytic cofactor, PQQ coordinates a metal ion (Ca^2+^ or a lanthanide) at the position occupied by the primary amide of K274 in the *E. coli* PqqU-PQQ structure (Fig. 5B). The presence of the shorter side chain glutamate at this position creates space capable of coordinating a metal ion. Moreover, glutamate is capable of coordinating a metal at this position, analogous to the PQQ-metal complexes observed in PQQ-dependent dehydrogenases (*1*, *6*–*8*). To test the hypothesis that this lysine-to-glutamate substitution may facilitate PqqU cobinding of a metal ion, we added one or more metal ions (Ca^2+^ or La^3+^) to the Chai-1 modeling. In most PqqU homologs with the glutamate substitution, Chai-1 modeled a Ca^2+^/La^3+^ ion at the hypothesized metal coordination, coordinated by both PQQ and the substituted glutamate side chain (Fig. 5B, fig. S9, and data S4). In some cases, a single Ca^2+^/La^3+^ ion was preferentially modeled in the semiconserved metal binding site identified in loop 6 of *E. coli* PqqU (fig. S10B). When a second metal ion was added to these predictions, it was modeled at the PQQ binding site. We observed analogous results whether Ca^2+^ or La^3+^ was modeled, suggesting that Chai-1 modeling does not readily distinguish between these ions. The pLDDT scores for metal ions in the PQQ predictions were generally low (data S4). However, we feel that the consistency of the modeling suggests that the lysine-to-glutamate substitution may facilitate cometal transport by PqqU. If this is the case, the fact that the lysine-to-glutamate substitution led to the inactivation of PqqU from *E. coli* is likely due to the need for additional substitutions to facilitate metal coordination and cotransport. In one of the modeled transporters, K274 was substituted for arginine, with the secondary amide of this side chain predicted to adopt an analogous position (Fig. 5B).

R536 in *E. coli* PqqU is important for PQQ import by this transporter but was less well conserved across the PqqU sequences we identified (fig. S7). In *E. coli* PqqU, R536 coordinates one of the carboxylic acid groups of PQQ (Fig. 5C). Analysis of the structural models of PqqU-PQQ complexes that lack arginine at this position indicated that in some cases, the coordination of this carboxylic acid group is compensated for by the presence of an arginine at a different location in the transporter (e.g., MBT5259169 from *Nitromaritima* sp.). Meanwhile, in other cases, coordination of the carboxylic acid group is not observed (e.g., QWK201176 from *Hydrogenobacter thermophilus*) (Fig. 5C). Considering that R536 is present in extracellular loop 8 of PqqU, it is involved in enclosing PQQ within the transporter. It may be important for the recognition of PQQ binding to trigger import, a function that may be compensated for by amino acid substitutions elsewhere in the protein.

### PQQ scavenging by PqqU is a conserved strategy

Most bacteria encoding PqqU were Gammaproteobacteria (*n* = 1346), which is present in 21 orders of this class. This is consistent with the abundance of genomes belonging to this class in available databases. Other notable PqqU-producing classes include Bacteroidota (*n* = 206), Gemmatimonadetes (*n* = 183), Acidobacteria (*n* = 17), Latescibacterota (*n* = 14), and Marinisomatota (*n* = 11) (Fig. 4A and fig. S8). PqqU was largely absent from Alphaproteobacteria (*n* = 1), even though these bacteria commonly contain PQQ-dependent quinoproteins and produce PQQ (*7*, *44*, *45*), indicating that they are likely primarily PQQ producers rather than scavengers, at least in the environments our genomic and metagenomic data originated from. However, this does not appear to be the predominant strategy adopted by PqqU-producing bacteria, as of the 22 phyla with members encoding PqqU, only 8 contain the machinery for PQQ biosynthesis. In total, the genes for PQQ biosynthesis could be identified in only 23% (*n* = 465) of PqqU producers and were absent from Bacteroidota (*n* = 206), Acidobacteria (*n* = 17), Latescibacterota (*n* = 14), Marinisomatota (*n* = 11), and Verrucomicrobia (*n* = 5), suggesting that bacteria from these phyla that produce PqqU rely exclusively on PQQ scavenging. When both PqqU and the PQQ biosynthesis operon were present, they generally did not show synteny, suggesting that they are regulated independently. However, we observed four Burkholderiales genomes showing synteny and the same gene orientation for these genes, indicating that co-regulation occurs in some organisms (fig. S11). Overall, this analysis indicates that the scavenging of PQQ from the environment at low concentrations using PqqU is a widespread strategy for the utilization of this cofactor.

PQQ is essential for the function of a diverse family of quinoprotein dehydrogenases, including lanthanide-dependent methanol dehydrogenases (XoxF clades), which are globally abundant and highly expressed in both soil and marine environments (*10*, *46*). While most PqqU producers we identified encode at least one predicted PQQ-dependent dehydrogenase (67%), no PQQ-dependent enzymes were identified in members of some Gammaproteobacterial orders/genera [Nitrosomonadaceae (*n* = 40), Ectothiorhodospirales (*n* = 8), and Arenimonas (*n* = 7)] and bacterial phyla Nitrospinota (*n* = 5) and Ignavibacteriota (*n* = 4). These bacteria also do not synthesize PQQ, suggesting an alternative role for the cofactor in these bacteria, possibly via a PQQ-dependent enzyme not identified in our search or as an antioxidant.

PqqU-producing bacteria are ecologically diverse, occurring in a wide range of aquatic, terrestrial, host-associated, built environments and extreme conditions (high and low pH and temperature). This indicates that PQQ is ubiquitous and chemically and thermally stable and is an important nutrient that contributes to the adaptability of bacteria to various environments (Fig. 4B). Gammaproteobacterial PqqU producers were found in all environment categories and are frequently capable of both producing and scavenging PQQ, suggesting that they play a key role in determining environmental PQQ concentrations. Acidobacteria and Bacteroidota phyla are ubiquitous and abundant members of soil microbiomes and are known to use quinoproteins (*47*, *48*). These phyla are well represented in public databases, but despite this, we only identified PqqU producers in class Vicinamibacteria (Acidobacteria) and classes Ignavibacteria, Rhodothermia, and Bacteroidia (Bacteroidota) that were primarily isolated from aquatic environments (Fig. 4B). Members of these phyla that do not encode PqqU may rely on endogenous PQQ production or soil PQQ concentrations that are high enough to satisfy their requirements by diffusion.

PqqU-producing bacteria also include important human, animal, and plant pathogens including multidrug-resistant *Pseudomonas aeruginosa*, *Klebsiella pneumoniae*, *Salmonella* sp., and *E. coli* (Fig. 4B and fig. S8). In members lacking PQQ biosynthesis machinery, PqqU may contribute to bacterial virulence by enabling metabolic versatility provided by quinoproteins (*1*), which would be beneficial in nutrient-limited environments inside the host or in environmental reservoirs. In *Salmonella* Serovar Typhi, PqqU (YncD) was reported to be important for virulence in a mouse intraperitoneal infection model. The attenuated Δ*pqqU* strain was used for vaccination and protected against subsequent infection with the wild-type strain (*49*). Subsequent work indicates that vaccination with recombinant PqqU from *Salmonella* Typhi also protects against its infection in a mouse model (*50*). If PQQ scavenging by PqqU is important for virulence, it suggests that it is an important nutrient for bacteria within animals or the human host. However, considering that animals do not synthesize PQQ, its presence in the body must result from dietary intake or synthesis by the gut microbiome (*51*). This would likely make PQQ more abundant in the gastrointestinal tract, although the high affinity of PqqU could make it compatible with scavenging trace PQQ present in human tissues. Further work is required to shed light on the possible role of bacterial PQQ import during infection. Last, the presence of PqqU in many pathogens suggests that it has potential as a cell surface receptor for engineered bacteriophage therapies, for example, the PqqU-dependent phage Isaaklselin in *E. coli* (*16*, *52*).

## DISCUSSION

**Fig. 6.**
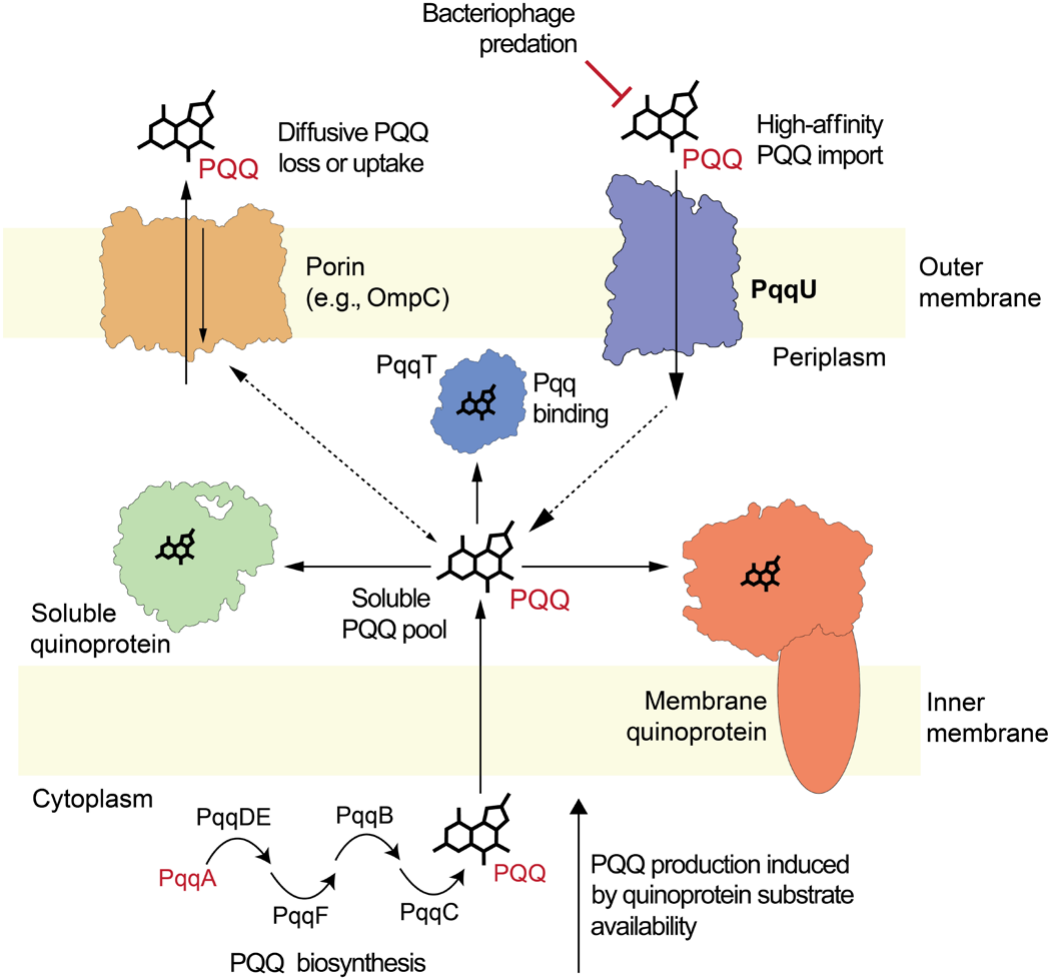
Model of the different strategies used by Gram-negative bacteria for obtaining PQQ for use in quinoproteins. This diagram is a composite of the different strategies used by Gram-negative bacteria to obtain PQQ. On the basis of our genomic analysis, some strains use both PQQ biosynthesis and scavenging by PqqU to obtain PQQ, while others rely on one of these strategies.

This work demonstrates that PqqU from Gram-negative bacteria binds PQQ with a high affinity, facilitating the import of low concentrations of this cofactor from the environment. By resolving the cryo-EM structure of 76-kDa PqqU in complex with PQQ at a high resolution of 1.99 Å, we show that PQQ is highly coordinated within an internal cavity of PqqU, which is formed by conformational changes of the transporter’s extracellular loops upon PQQ binding. This conformational change traps PQQ inside the transporter, blocks the binding of a PqqU-dependent bacteriophage, and may facilitate a double-gated import mechanism that prevents the uptake of unwanted compounds. This work also confirms that PqqU transports a non–metal-containing substrate, which is unusual for a TBDT and underpins the importance of PQQ as an external nutrient for Gram-negative bacteria (*26*). Using the knowledge of the PQQ binding site revealed by our cryo-EM structure and functional analysis, we show that PqqU facilitates PQQ import in Gram-negative bacteria from 22 phyla, which are present in diverse environments. This indicates that PQQ is an important externally derived nutrient and a ubiquitous common good of diverse microbiomes. The presence of PqqU and quinoproteins in many bacteria that lack the machinery for PQQ biosynthesis indicates that PQQ scavenging is a widespread strategy for satisfying metabolic requirements for this cofactor, which may be used in conjunction with PQQ production and sequestration to prevent its loss from the cell via diffusion (Fig. 6).

## MATERIALS AND METHODS

### Bacterial strains and growth conditions

For cloning, *E. coli* DH5α were maintained on Lysogeny Broth (LB) agar plates and broth [tryptone (10 g/liter), yeast extract (5 g/liter), NaCl (5 g/liter), and agar (15 g/liter)] with ampicillin (100 μg/ml), where appropriate. Cultures were incubated at 37°C with orbital shaking at 180 rpm.

For expression of 10×His-PqqU, *E. coli* BL21 (DE3) C41 cells were transformed with the plasmid pET-20b-pqqU. A starter culture was inoculated with 90% of the transformation and grown in LB [ampicillin (100 μg/ml)] overnight at 37°C with rotary shaking at 180 rpm. The remaining 10% were plated as contamination control. Main cultures were inoculated with the starter culture to a start OD at 600 nm (OD_600_) of 0.1 and grown until an OD_600_ of 1 in TB broth [tryptone (12 g/liter), yeast extract (24 g/liter), K_2_HPO_4_ (12.3 g/liter), and KH_2_PO_4_ (2.52 g/liter)] at 37°C at 180 rpm. Cultures were cooled to 4°C after which the expression was induced with 0.3 mM isopropyl-β-d-thiogalactopyranoside. Cultures were grown for pqqU expression for ~18 hours at 24°C at 180 rpm.

For complementation experiments, *E. coli* BW25113 Δ*pts*Δ*pqqU* starter cultures were grown in LB containing 0.05% l-arabinose and ampicillin (100 μg/ml), where appropriate. Complementation main cultures were grown in M63 media [14.13 mM (NH_4_)_2_SO_4_, 38.95 mM KH_2_PO_4_, 1 mM MgSO_4_, 3.3 μM FeSO_4_, and 0.05% arabinose, pH 7] and, where appropriate, 0.1% glucose and 10 nM PQQ. Main cultures were inoculated with a start OD_600_ of 0.05 after resuspending the starter culture cell pellet in M63 media and 0.05% l-arabinose.

### Generation of PqqU mutants by site-directed mutagenesis

Binding site mutations of PqqU were generated from the plasmid template pBAD24-pqqU using the Q5 Site-Directed Mutagenesis Kit (New England BioLabs) according to the manufacturer’s instructions. Mutations were confirmed by nanopore sequencing (Primordium Labs). See table S4 for primer sequences.

### Complementation growth experiments of PqqU mutants

Growth curves were generated from a total of four biological and two technical replicates (five growth curves in total). Starter cultures were prepared as previously described above, grown overnight, and kept on ice during the day. Two hours before inoculation of the main cultures, starter cultures were centrifuged at 4500*g* for 10 min and resuspended in M63 media containing 0.05% l-arabinose. Complementation cultures were prepared as described above. Complementation cultures were grown in M63 media providing 0.1% glucose as an energy/carbon source and 10 nM PQQ as the cofactor for Gcd activity. Cells required a 10-hour equilibration time before we could measure the growth for 14 hours. Growth curves were plotted and analyzed with GraphPad Prism 10.0.3 software. See tables S5 and S6 for a list of plasmids and strains used for these experiments.

### Comparison of the PqqU presence by immunoblotting

The cultures were grown according to the PqqU complementation growth experiment conditions. After 24 hours of growth, the culture volumes were harvested by equal cell mass. Cells were lysed and centrifuged at 13,000 rpm to separate cell debris from membranes and soluble proteins. Equal volumes of the supernatant were loaded on a 4 to 12% SDS–polyacrylamide gel electrophoresis (SDS-PAGE) gel. Proteins were transferred to a polyvinylidene difluoride membrane (Immobilon-P, 0.45 μm) with a Trans-Blot Turbo (Bio-Rad). After protein transfer, the membrane was blocked with 5% bovine serum albumin in TBST [20 mM tris-HCl (pH 7.5), 150 mM NaCl, and 0.1% Tween 20] for 1 hour, followed by 1-hour incubation with a horseradish peroxidase–conjugated anti-His antibody (Abcam). The polyvinylidene difluoride membrane was then washed four times for 10 min with TBST. His-tagged PqqU was detected with a Bio-Rad ChemicDoc Imaging System.

### PqqU purification

PqqU (formerly YncD) was purified from *E. coli* C41 containing pET-20b-pqqU on eight occasions from either 8 or 16 liters of LB media per purification. The yield of PqqU was 150 μg/liter on average depending on pellet size and which detergent was used for membrane solubilization. Cell pellets were resuspended in a final volume of 100 ml of lysis buffer [50 mM tris-HCl (pH 7.4), 150 mM NaCl, 10 mM imidazole, 2 mM MgCl_2_, lysozyme (0.1 mg/ml), deoxyribonuclease (0.05 mg/ml), and 1× protease inhibitor] and homogenized with a glass homogenizer before being subjected to cell lysis in a high-pressure cell disruptor. The lysate was centrifuged at 27,000*g* for 20 min at 4°C. The resulting supernatant was used for ultracentrifugation at 100,000*g* for 40 min at 4°C. The membrane pellet was resuspended in 40 ml of solubilization buffer [50 mM tris-HCl (pH 7.4), 150 mM NaCl, 10 mM imidazole, and 5% Elugent detergent] and homogenized in a glass homogenizer. Solubilization was done at room temperature with slow rotary shaking. The sample was centrifuged at 27,000*g* for 20 min, and the resulting supernatant was loaded two times on a HisTrap HP 5-ml affinity column. After extensive washing with wash buffer [50 mM tris-HCl (pH 7.4), 500 mM NaCl, 10 mM imidazole, and 0.03% *n*-dodecyl-β-d-maltoside (DDM) or 0.025% LMNG], PqqU was eluted with a step gradient in elution buffer [50 mM tris-HCl (pH 7.4), 150 mM NaCl, 1 M imidazole, and 0.03% DDM or 0.025% LMNG]. When purified in DDM, PqqU eluted in 250 mM imidazole, while it eluted in 500 mM Imidazole when purified in LMNG. Elution fractions were analyzed via SDS-PAGE and pooled and concentrated to a volume of 5 ml in an Amicon Ultra-15 Centrifugal Filter 30-kDa MWCO Millipore. Size exclusion chromatography was done with a Superdex HiLoad 200 pg 26/600 in size exclusion buffer [50 mM tris-HCl (pH 7.4), 150 mM NaCl, and 0.03% DDM or 0.025% LMNG]. PqqU eluted in a single peak at a retention volume of 140 to 180 ml when purified in DDM and in a double peak at a retention volume of 140 to 180 ml. PqqU could be concentrated to up to 15 mg/ml without much loss of protein in an Amicon Ultra-15 Centrifugal Filter 100-kDA MWCO Millipore and stored at −80°C without glycerol. Before cryo-EM and ITC, 20 to 100 μl of PqqU (purified in LMNG) was diluted in 50 ml of size exclusion buffer without detergent and reconcentrated to remove excess detergent.

### SDS-PAGE analysis

Protein samples were run on a Bolt 4 to 12% SDS-PAGE gel according to the manufacturer’s instructions. Gel staining was done with AcquaStain Protein Gel Stain (Bulldog) and subsequently washed in distilled H_2_O. Protein samples were generally loaded by constant volume.

### Cryo-EM imaging and data processing

Cryo-EM imaging was performed on PqqU, which was initially purified in DDM and then detergent exchanged to LMNG with subsequent removal of excess LMNG, as described above. For grid preparation, UltrAuFoil gold grids (Quantifoil GmbH) (*53*) were glow discharged at 30 mA for 60 s (PELCO easiGlow) in air. Three microliters of the sample was applied at a concentration of 43 μM PqqU (3.3 mg/ml) with 910 μM PQQ (dissolved in H_2_O) to the glow-discharged grids. The excess protein solution was blotted off using a blot force of 0 and a blot time of 3 s in a Mark IV vitrobot (Thermo Fisher Scientific), which was maintained at 100% humidity and 4°C.

Imaging was performed on a G1-Titan Krios (Thermo Fisher Scientific) with a Schottky field emission gun operated at 300 kV and equipped with a Gatan K3 mounted on a Gatan BioQuantum energy filter. The fast data collection strategy in Electron Microscopy Public User (EPU) (Thermo Fisher Scientific) enabled with aberration-free image-shift alignments was used for automated data collection. Zero-loss energy–filtered images were acquired at a nominal magnification of 105,000× using a narrow energy filter slit width of 10 eV. K3 was operated in counting CDS mode with a pixel size of 0.82 Å. Movies were collected with a total dose of 60 e^−^ Å^−2^ accumulated over 5.22-s exposure time at a dose rate of 8.518 e^−^ pixel^−1^ s^−1^ fractionated into 60 frames. A total of 8744 movies was recorded.

For image processing, the resultant movies were dose weighted and motion corrected using UCSF Motioncor2 (version 1.6.3) (*54*) to output both dose-weighted and non–dose-weighted averages. The non–dose-weighted averages were used for estimating contrast transfer function (CTF) parameters using CTFFIND 4.1.8 (*55*) using RELION 4.0.1 (*56*) wrapper. Particle picking was performed using Gautomatch (0.53) (https://gitlab.psi.ch/bliven_s/buildblocks/-/tree/master/EM/Gautomatch) on the full dataset using a blob diameter of 80 Å. This resulted in 2,112,379 particles from resultant coordinates, which were extracted and binned four times to a box size of 64 pixels. These particles were imported to cryoSPARC (version 4.2.0) (*57*) and subjected to 2D classification to retain 482,616 particles. Ab initio classification with class similarity of 0 and 3 classes was performed to result in 258,422 particles, giving a consensus ab initio model that had features of micelle-associated PqqU. 3D refinement in cryoSPARC 4.2.0 was performed to further center the particles. The coordinates were then exported back to RELION using pyem version 0.5 (*58*) and reextracted and unbinned in RELION 4.0.1 centered on the refined coordinates. The particles were reimported back to cryoSPARC 4.2.0. One round of 2D classification resulted in retaining 252,169 particles for further processing. A round of homogeneous refinement followed by two rounds of nonuniform (NU) refinement (*59*) with defocus, tilt, and trefoil refinement (*59*, *60*) resulted in a 2.69-Å map. These particles were then subjected to Bayesian polishing (*61*) in RELION 4.0.1, and the resultant polished particles were then subjected to a round of homogeneous refinement followed by two rounds of NU refinement to yield a 2.49-Å map. Furthermore, baited heterogeneous refinement with two classes, with one of them being a noisy reconstruction, was used to filter away junk particles. The filtered particles were then subjected to further NU refinement and heterogeneous refinement to retain 148,172 particles, resulting in a 2.29-Å-resolution map. To push the resolution further, these particles were used to train the TOPAZ particle picker (*62*) on the full dataset. The trained model was then able to pick a total of 6,488,281 particles. These were then subjected to the same processing workflow as above. A round of 2D classification resulted in retaining 1,179,837 particles. These were then subjected to a round of homogeneous refinement followed by baited heterogeneous refinement to yield 872,884 particles. Further NU refinement on the resultant particles along with CTF refinement yielded a 2.18-Å-resolution map. These particles were then subjected to Bayesian polishing in RELION 4.0.1 and were further subjected to NU refinement with CTF refinement to yield a 2.06-Å-resolution map. A final round of local refinement with a micelle-subtracted map [generated using UCSF Chimera (version 1.16) (*47*)]–derived mask resulted in the final 1.99-Å-resolution map (gold standard FSC 0.143 criteria).

### PqqU + PQQ model building and visualization

The apo crystal structure of PqqU was used for docking into the 1.99-Å-resolution map of PqqU using ChimeraX 1.6.1. Model building was done using WinCoot CCP4 0.9.8.1. The structural coordinates for PQQ were extracted from PDB entry 7WMK and used to fit in the experimental maps. PHENIX was used for real-space refinement, and DOUSE was used for initial identification of H_2_O but had to be heavily corrected. Model quality was assessed and confirmed using the PDB validation web tool. Images were created using ChimeraX and PyMOL.

### Isothermal titration calorimetry

PqqU for ITC was purified in LMNG as described previously. On the day before the ITC experiment, detergent was removed from the sample by dilution in 30 ml of detergent-free buffer and concentration to ~150 μl. This was possible due to the high affinity of LMNG to hydrophobic protein surfaces and done to prevent the interference of excess detergent during the ITC measurement. Afterward, the sample was dialyzed in a 3.5-kDa cutoff dialysis bag (Spectra/Por Dialysis Membrane 3.5 kD) in 2 liters of dialysis buffer [50 mM tris-HCl (pH 7.4) and 150 mM NaCl] at 4°C overnight on a magnetic stirrer on low speed. Dialyzed PqqU was recovered and centrifuged at 13,000*g* for 10 min at 4°C. The PqqU concentration was measured after centrifugation, and PQQ (methoxatin disodium salt) was dissolved in the same overnight dialysis buffer to a stock concentration of 5 mM PQQ. ITC was performed using the Malvern MicroCal PEAQ ITC. The three technical replicates and controls were performed from the same purified PqqU sample. PQQ (100 μM) was titrated into 20 μM PqqU over 40 min in 19 injections of 2 μl. Graphs and figures were generated from baseline-corrected data in GraphPad Prism 10.0.3 software.

### AlphaFold2 structural modeling

AlphaFold2 multimer modeling predictions were run using AlphaFold version 2.3.2 through the University of Melbourne Spartan computing cluster (*33*, *34*). Sequences for *E. coli* PqqU, IsaakIselin phage SIP-1 (QXV80559), RBP-1 (QXV80560), and RBP-2 (QXV80561) were modeled alone and in various combinations to assess complex formation. The top five ranked models from each run were interrogated for consistency and model quality (pLDDT, pTM, and predicted aligned error scores), and the top-ranked model was visualized using PyMOL (Schrödinger).

### Chai-1 structural modeling

Chai-1 structural modeling was performed using the Chai-1 web service, using default settings without the use of multiple sequence alignments (https://lab.chaidiscovery.com/dashboard) (*41*). The single-letter amino acid sequence of PqqU homologs was provided to Chai-1, along with PQQ and either La^2+^ or Ca^2+^ in SMILES string format. Initially, the PqqU homolog was modeled with a single metal ion and PQQ molecule. If PQQ binding was observed in PqqU outside the binding site, a second PQQ molecule was included. For PqqU homologs with a glutamate residue at the equivalent position to K274 of *E. coli* PqqU, a second metal ion was modeled if the first metal ion bound to the semiconserved binding site in loop 6 of the PqqU homolog. The top five ranked models from each run were interrogated for consistency and model quality (pLDDT, pTM, and predicted aligned error scores), and the top-ranked model was visualized using PyMOL (Schrödinger).

### Bioinformatic analysis

The National Center for Biotechnology Information NR GenBank release 254 (15 February 2023) database was queried using blastp v2.14.1+ for PqqU (NP_415968.1) via the command line with an e-value cutoff of 1 × 10^−5^. Sequences were also retrieved from the UniProt reference proteome database using an e-value cutoff of 1 × 10^−5^. Retrieved sequences were aligned using FAMSA (fast and accurate multiple sequence alignment) version 1.2.5 (*63*) with default parameters and trimmed using trimal version 1.4.1 and the -gappyout option (*64*). Sequences were manually verified on the basis of the presence of conserved PqqU substrate binding residues Y99 (or F99 or W99), R304, and R365 using Geneious (*65*), resulting in 16,220 nonredundant PqqU homologs. BioSample IDs from verified PqqU homologs were used to retrieve 4040 genomes (most retrieved PqqU sequences were associated with metagenomes) from the BV-BGC database (*60*). To account for any database inconsistencies, genomes were again searched and manually verified to contain PqqU homologs before being dereplicated using dRep 3.4.2 with default settings (*66*), resulting in a set of 1861 genomes. Genomes were searched for the PQQ biosynthesis operon (PqqA, PqqB, PqqC, PqqD, PqqE, and PqqF) from *P. aeruginosa* using cblaster version 1.3.18 (*67*). PqqU (NP_415968.1) was also used as a query to determine whether it is colocalized with the PQQ biosynthesis operon. The colocalization and orientation of the genes were visualized using clinker version 0.0. (*68*). PQQ-dependent dehydrogenases were identified using a hidden Markov model profile taken from Diamond *et al.* (*48*). PQQ protein domains were predicted in the retrieved sequences by using hmm search (version 3.3) (*69*) to search against the protein family database (version 36.0) (*70*) for PQQ pfams PF01011, PF13360, and PF13570 with an e-value cutoff of 1 × 10^−5^. A ribosomal protein tree was built using a concatenation of 16 ribosomal proteins identified in each genome using GOOSOS.py (https://github.com/jwestrob/GOOSOS). The following hidden Markov models were used: Ribosomal_L2 (K02886), Ribosomal_L3 (K02906), Ribosomal_L4 (K02926), Ribosomal_L5 (K02931), Ribosomal_L6 (K02933), Ribosomal_L14 (K02874), Ribosomal_L15 (K02876), Ribosomal_L16 (K02878), Ribosomal_L18 (K02881), Ribosomal_L22 (K02890), Ribosomal_L24 (K02895), Ribosomal_S3 (K02982), Ribosomal_S8 (K02994), Ribosomal_S10 (K02946), Ribosomal_S17 (K02961), and Ribosomal_S19 (K02965). Ribosomal S10 model PF00338 was also used for the identification of Chloroflexi. All trees were constructed and decorated using iTOL (*71*).
