# Supplementary material for: High-affinity PQQ import is widespread in Gram-negative bacteria
Source: Sci Adv. 2025 May 30;11(22):eadr2753. doi: 10.1126/sciadv.adr2753 (PMC12124388; doi:10.1126/sciadv.adr2753)
Supplement: Supplementary file 1 — Figs. S1 to S11 Tables S1 to S6 Legends for movies S1 to S3 Legends for data S1 to S4 [file sciadv.adr2753_sm.pdf]

Science Advances
AAAS

Supplementary Materials for

High-affinity PQQ import is widespread in Gram-negative bacteria

Fabian Munder *et al.*

Corresponding author: Rhys Grinter, rhys.grinter@unimelb.edu.au; Hari Venugopal, hari.venugopal@monash.edu

Sci. Adv. **11**, eadr2753 (2025)
DOI: 10.1126/sciadv.adr2753

The PDF file includes:

Figs. S1 to S11
Tables S1 to S6
Legends for movies S1 to S3
Legends for data S1 to S4

Other Supplementary Material for this manuscript includes the following:

Movies S1 to S3
Data S1 to S4

SUPPLEMENTAL FIGURES

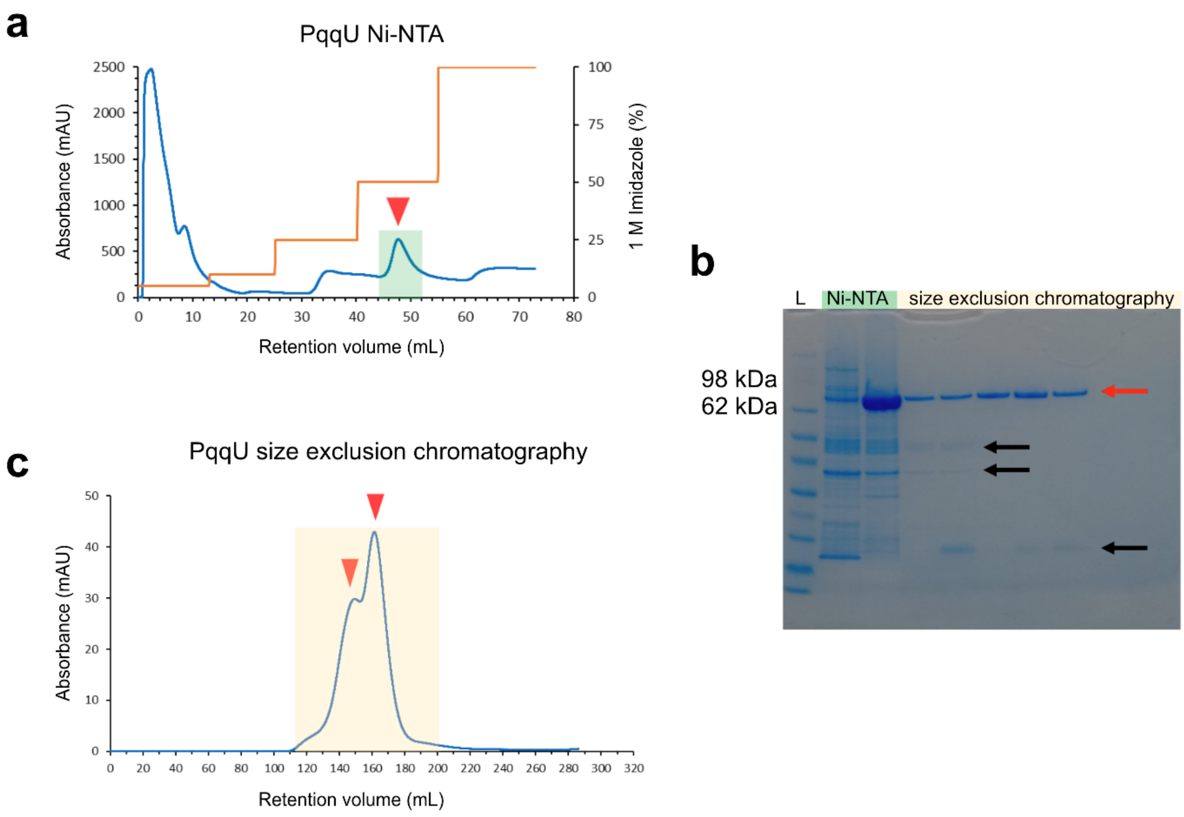

Figure S1: Purification of PqqU in LMNG. a, the elution profile (Blue) of PqqU from HisTrap HP 5-ml with a step gradient of Imidazole (Orange). The red arrow indicates the elution peak corresponding to PqqU. **b**, elution profile of PqqU during size exclusion chromatography off a Superdex HiLoad 200pg 26/600 column. Red arrows indicate the double peak corresponding to PqqU when purified in the presence of LMNG. **c**, SDS-PAGE gel of affinity chromatography and size exclusion chromatography fractions corresponding to the observed double peak of the chromatogram. The red arrow indicates the band corresponding to PqqU (76 kDa), and the black arrows highlight minor contaminating proteins. Ni-NTA = Nickel-Nitrilotriacetic Acid.

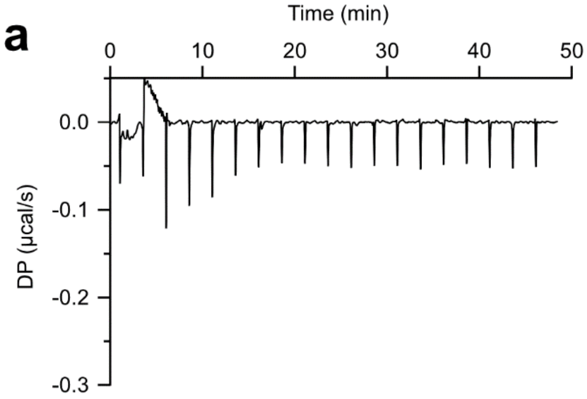

Figure S2: ITC buffer control injected into PqqU. a, buffer control of PqqU+PQQ isothermal titration calorimetry experiment. Dialysis buffer was injected into 20 µM PqqU over ~50 minutes in 19 injections of 2 µl, the first injection is 0.4 µl. Experiments were conducted in triplicate on a Malvern MicroCal PEAQ-ITC and analysed with the accompanying software. Baseline-corrected data were plotted with GraphPad Prism. DP = Differential Power.

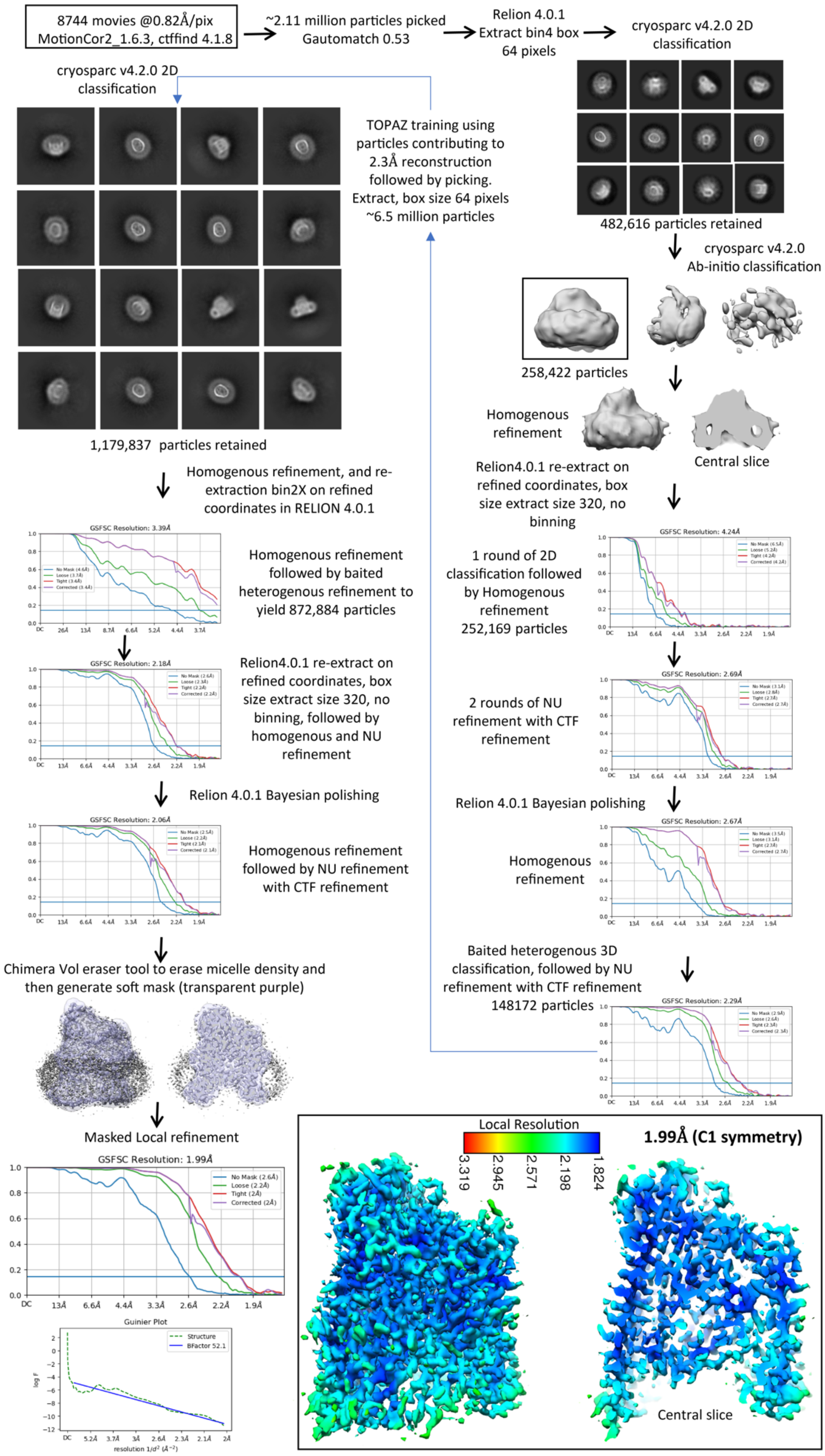

Figure S3: CryoEM data processing workflow for PqqU.

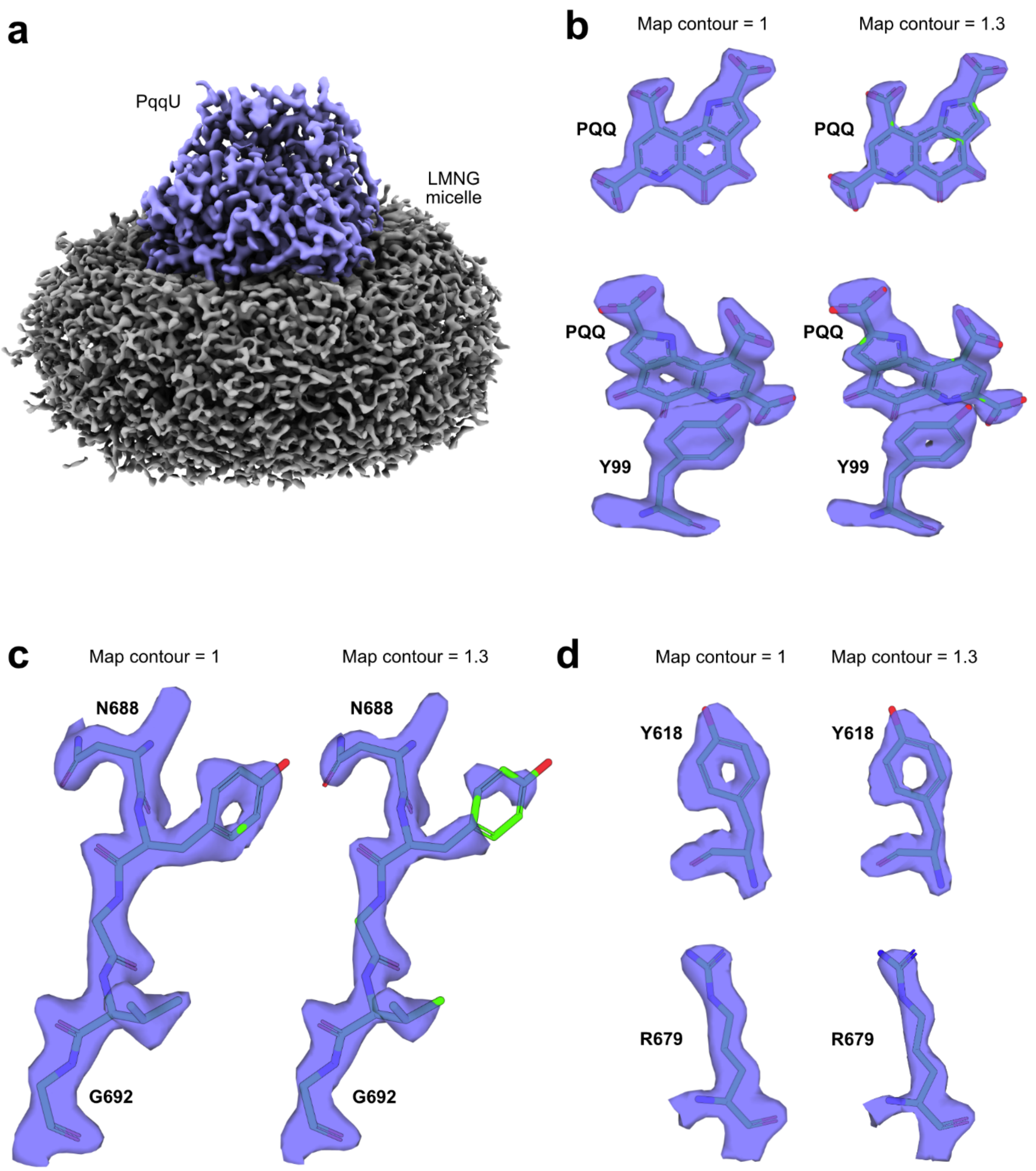

Figure S4: Additional structural images/analysis of PqqU and the PqqU-PQQ complex. a, Coulomb potential map of PqqU (Light-blue, contour 0.65) in its LMNG detergent micelle (Grey, contour 0.17). Map visualized with ChimeraX 1.7.1. **b-d**, close-up comparison of Coulomb potential maps of PQQ and selected residues at contours 1 and 1.3. Maps visualized with PyMOL (Schrödinger). LMNG = Lauryl Maltose Neopentyl Glycol.

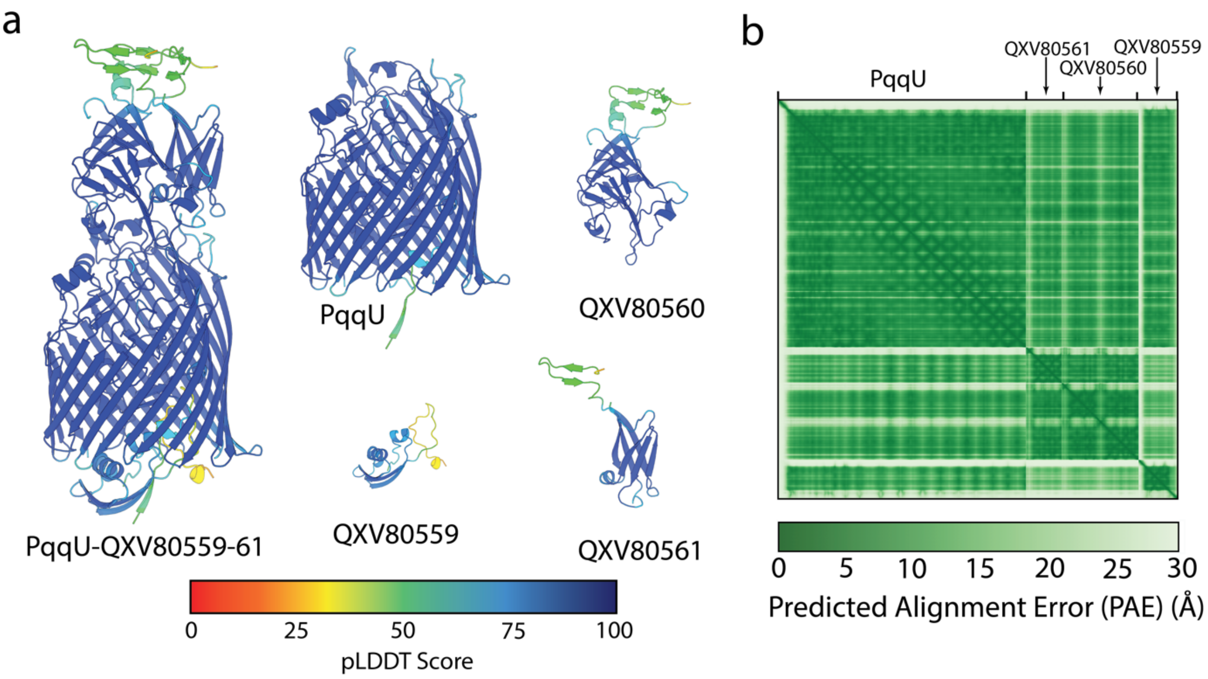

Figure S5: Confidence scores for the AlphaFold2 model of the PqqU-IsaakIselin Phage receptor binding protein complex. a, the PqqU and phage binding proteins (QXV80560-62) are coloured by pLDDT confidence score. **b**, 2D matrix of predicted alignment error (PAE) for the PqqU-QXV80560-61 structure.

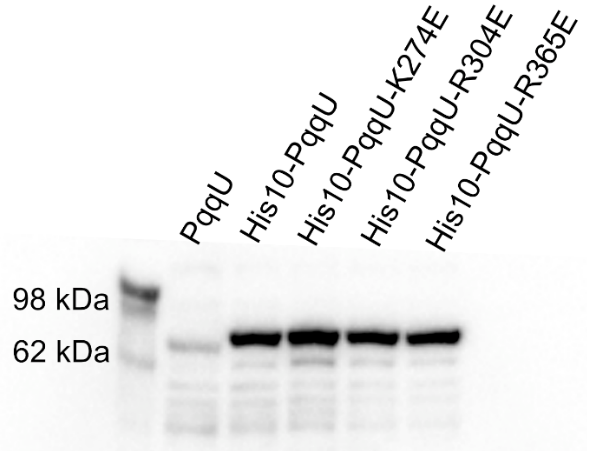

Figure S6: Western blot of PqqU expression levels. His10-tagged PqqU and relevant PqqU binding site mutants were overexpressed in *E. coli ΔptsΔpqqU* and grown for 24 h. All samples were normalized by cell mass and probed with an anti-His antibody. The wildtype PqqU and the 3 growth-defect mutants (K274E, R304E, and R365E) show comparably intense signals at the expected molecular weight (~76 kDa), which indicates similar expression levels.

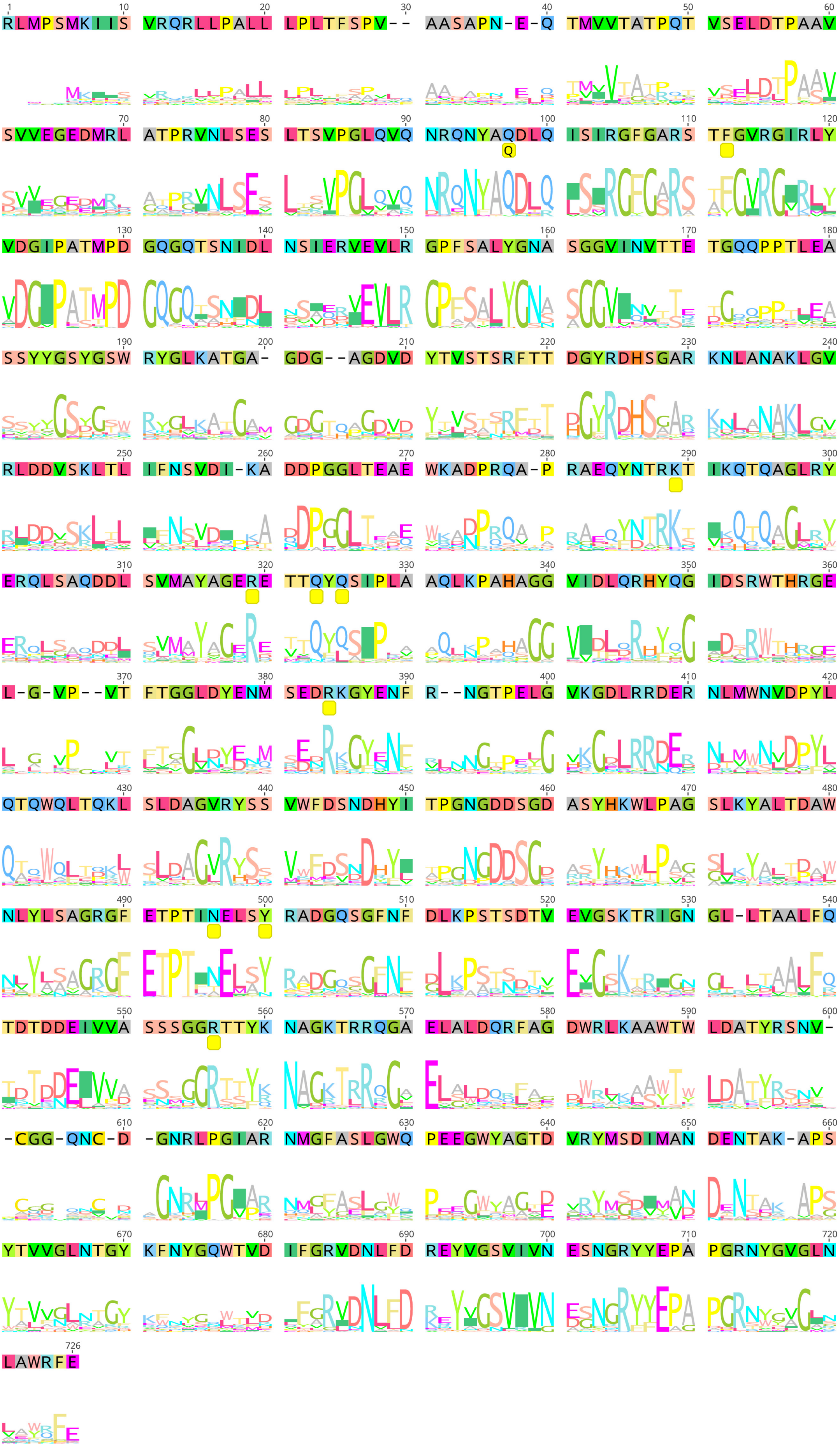
RLMPSMKIIS VRQRLLPALL LPLTFSPV-- AASAPN-E-Q TMVVTATPQT VSELDTPAAV
SVVEGEDMRL ATPRVNLSES LTSVPGLQVQ NRQNYAQDLQ ISIRGFGARS TFGVRGIRLY
VDGIPATMPD GQGQTSNIDL NSIERVEVLR GPFSALYGNA SGGVINVTTE TGQQPPTLEA
SSYYGSYGSW RYGLKATGA- GDG--AGDVD YTVSTSRFTT DGYRDHSGAR KNLANAKLGV
RLDDVSKLTL IFNSVDI-KA DDPGGLTEAE WKADPRQA-P RAEQYNTRKT IKQTQAGLRY
ERQLSAQDDL SVMAYAGERE TTQYQSIPLA AQLKPAHAGG VIDLQRHYQG IDSRWTHRGE
L-G-VP--VT FTGGLDYENM SEDRKGYENF R--NGTPELG VKGDLRRDER NLMWNVDPYL
QTQWQLTQKL SLDAGVRYSS VWFDSNDHYI TPGNGDDSGD ASYHKWLPAG SLKYALTDAW
NLYLSAGRGF ETPTINELSY RADGQSGFNF DLKPSTSDTV EVGSKTRIGN GL-LTAALFQ
TDTDDEIVVA SSSGGRTTYK NAGKTRRQGA ELALDQRFAG DWRLKAAWTW LDATYRSNV-
-CGG-QNC-D -GNRLPGIAR NMGFASLGWQ PEEGWYAGTD VRYMSDIMAN DENTAK-APS
YTVVGLNTGY KFNYGQWTVD IFGRVDNLFD REYVGSVIVN ESNGRYYEPA PGRNYGVGLN
LAWRFE

Figure S7: A consensus sequence logo plot shows amino acid conservation across the PqqU homologues identified in our search. The consensus logo was generated using Geneious from the full PqqU homolog alignment containing 16,220 sequences aligned with FAMSA and trimmed using TrimAL (Supplemental Data S3). PQQ-interacting residues are indicated by a yellow box. Residue graphs illustrate amino acid conservation in PqqU homologs. The majority consensus is displayed above the residue graphs.

This figure is available via Figshare at: 10.6084/m9.figshare.28862273

Figure S8: Horizontal genome tree of PqqU producers. A phylogenetic tree constructed from a concatenated alignment of 16 ribosomal proteins. The tree includes 1,861 genomes where 8 or more ribosomal proteins were identified. Genome names are based on their GTDB-Tk classification found in.

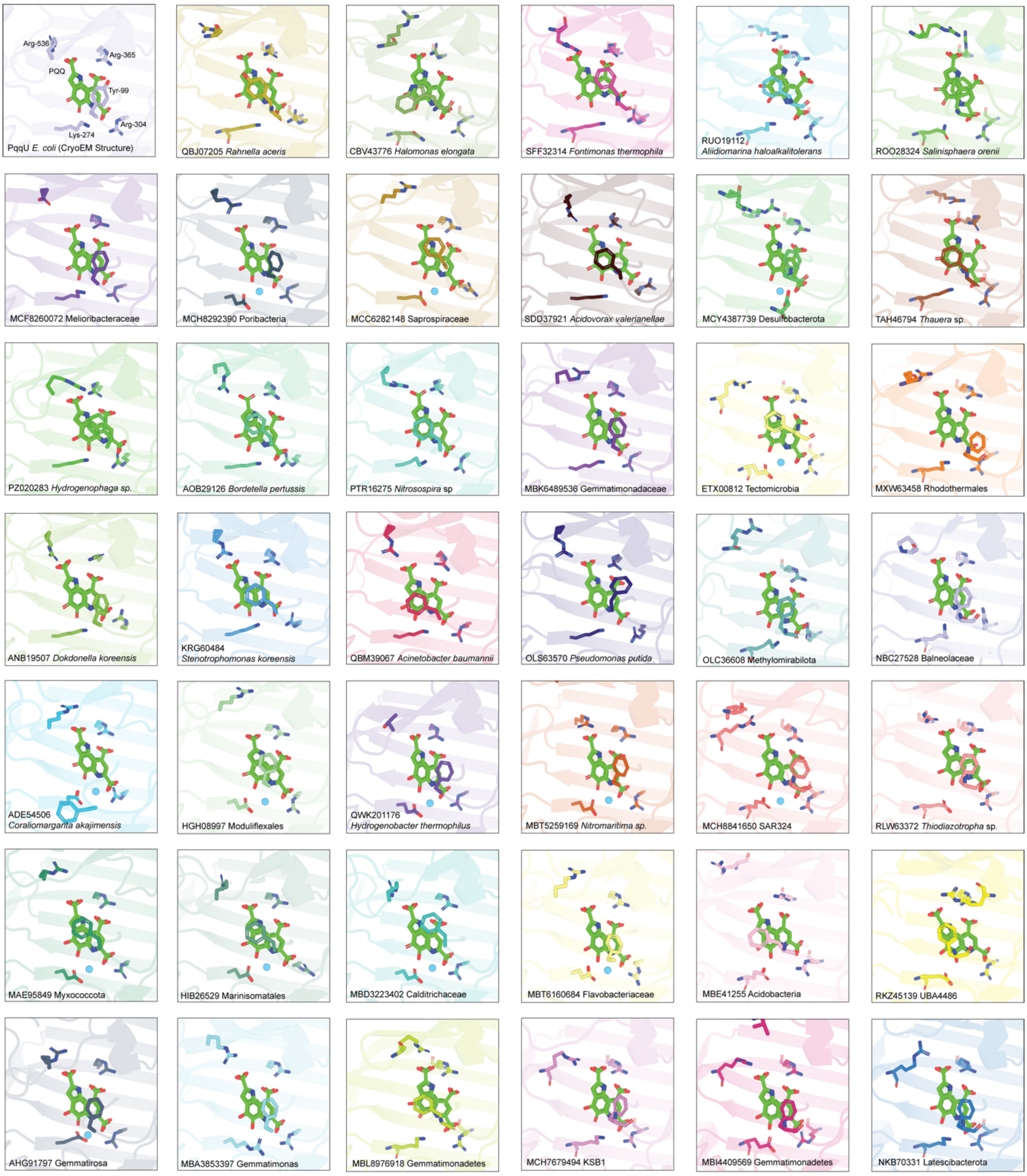

Figure S9. The Chai-1 predicted PQQ-binding site of PqqU homologs representing the diversity of sequences identified in phylogenetic analysis. A zoomed-in view of the PQQ binding site of the experimental structure of the PqqU-PQQ complex from *E. coli* and Chai-1 models of diverse PqqU sequences. The protein structure is presented as a transparent cartoon, and PQQ as well as key binding residues, are shown as sticks. When present metal ions are shown as light blue spheres (see Supplemental Data S4 for coordinate files for all models).

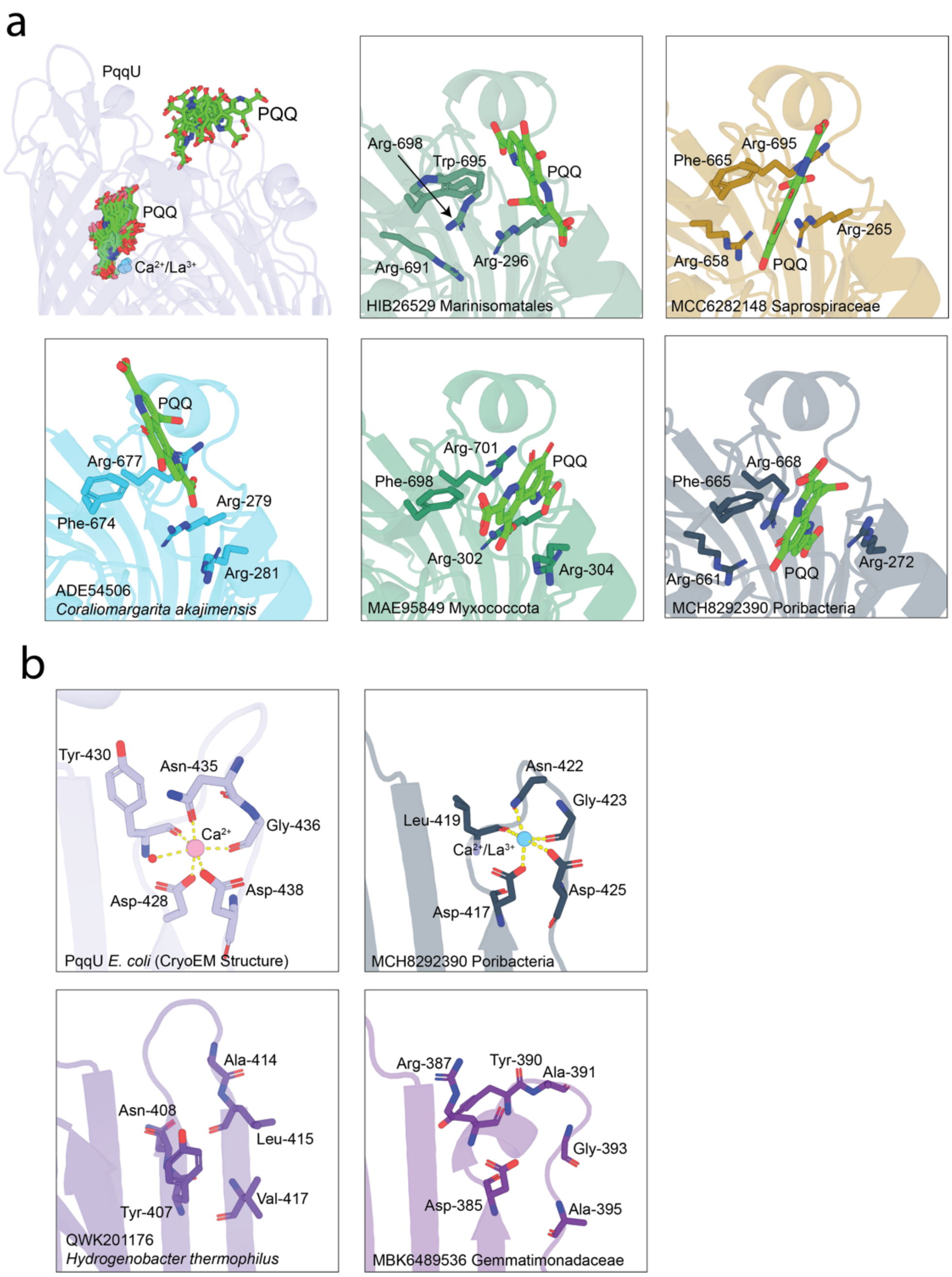

Figure S10: Additional structural features of PqqU-PQQ complex models. a, Identification of a possible secondary PQQ binding site on the surface of some Chai-1 PqqU models. For a number of PqqU sequences, modelling with a single PQQ molecule led to placement at a semiconserved site on the external surface of the transporter; when a second PQQ molecule was added, it was modelled in the canonical PQQ binding site. This figure shows PQQ modelled into the external binding site for several models. The protein structure is presented as a transparent cartoon, and PQQ as well as key binding residues are shown as sticks. **b**, a comparison of the semi-conserved metal binding site in loop 6 of *E. coli* PqqU, with selected Chai-1 models of PqqU.

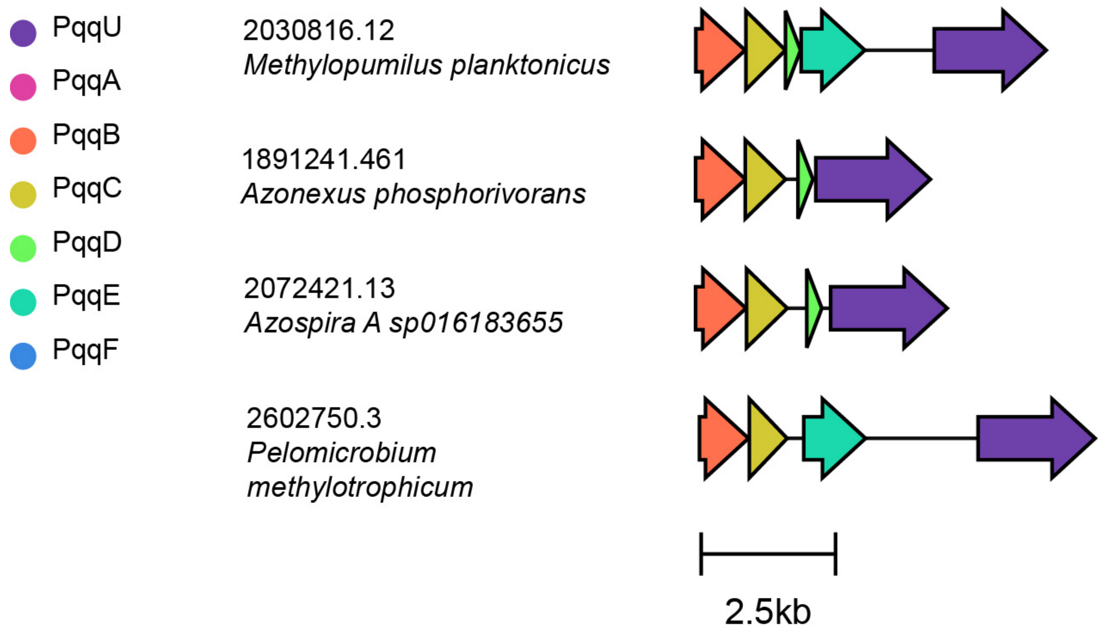

Figure S11: Synteny and orientation of *pqqU* and the PQQ biosynthesis operon in four *Burkholderiales* genomes.

SUPPLEMENTAL TABLES

Table S1: ITC-derived PqqU-PQQ binding parameters. ITC experiment output for three replicate runs and the buffer control. Output generated by the MicroCal Analysis software.

Sample Name	Temp	[Syr] (M)	[Cell] (M)	Ligand in Cell	N (sites)	KD (M)	ΔH (kcal/mol)	ΔG (kcal/mol)	-TΔS (kcal/mol)	Red. Chi-Sqr. $(kcal/mol)^2$
1st run	25.1	1.00E-04	2.00E-05	No	0.712	1.00E-12	-14.4	-16.4	-1.94	13.2
2nd run	25.1	1.00E-04	2.00E-05	No	0.743	1.00E-12	-15	-16.4	-1.39	7.37
3rd run	25.1	1.00E-04	2.00E-05	No	0.775	1.00E-12	-13.7	-16.4	-2.66	2.77
Buffer control	25.1	N/A	N/A	N/A	N/A	N/A	N/A	N/A	N/A	N/A

Table S2: Cryo-EM data collection, refinement and validation statistics. All datasets were collected with a zero-loss filtering slit width of 10 eV and with 60 frames per movie.

	PqqU-PQQ (EMD- 45192) (PDB 9C4O)
**Data collection and processing**	
Magnification	105,000X
Voltage (kV)	300
Electron exposure (e–/Å^2)	60
Defocus range (μm)	0.4-1.2
Pixel size (Å)	0.82
Symmetry imposed	C1
Initial particle images (no.)	6,488,281
Final particle images (no.)	872,884
Map resolution (Å)	1.99
FSC threshold	0.143
Map resolution range (Å)	1.84-3.34
**Refinement**	
Initial model used (PDB code)	6V81
Model resolution (Å)	1.99
FSC threshold	0.143
Model resolution range (Å)	1.824 – 3.319
Map sharpening *B* factor (Å^2)	52.1
Model composition	
Non-hydrogen atoms	5147
Protein residues	661
Ligands	1
B factors (Å^2)	
Protein	5.24 – 60.6
Ligand	13.39 – 30.5
R.m.s. deviations	
Bond lengths (Å)	0.32
Bond angles (°)	0.57
Validation	
MolProbity score	1.04
Clashscore	6
Poor rotamers (%)	1
Ramachandran plot	
Favored (%)	98
Allowed (%)	2
Disallowed (%)	0

Table S3: PqqU homologs modelled in complex with PQQ using Chai-1.

GTDB-tk taxa	Protein Accession	Model pTM score	Model piTM score	Model number
d__Bacteria;p__Pseudomonadota;c__Gammaproteobacteria;o__Burkholderiales;f__Burkholderiaceae_B;g__Acidovorax_A;s__Acidovorax_A valerianellae	SDD37921.1	0.88	0.72	1
d__Bacteria;p__Pseudomonadota;c__Gammaproteobacteria;o__Burkholderiales;f__Burkholderiaceae_B;g__Hydrogenophaga;s__Hydrogenophaga sp003241965	PZO20283.1	0.9	0.53	2
d__Bacteria;p__Pseudomonadota;c__Gammaproteobacteria;o__Burkholderiales;f__Burkholderiaceae_C;g__Bordetella;s__Bordetella pertussis	AOB29126.1	0.91	0.72	3
d__Bacteria;p__Pseudomonadota;c__Gammaproteobacteria;o__Burkholderiales;f__Rhodocyclaceae;g__Thauera;s__Thauera sp004295105	TAH46794.1	0.91	0.57	4
d__Bacteria;p__Pseudomonadota;c__Gammaproteobacteria;o__Burkholderiales;f__Nitrosomonadaceae;g__Nitrosospira;s__Nitrosospira sp003050965	PTR16275.1	0.89	0.73	5
d__Bacteria;p__Verrucomicrobiota;c__Verrucomicrobiae;o__Opitutales;f__DSM-45221;g__Coraliomargarita;s__Coraliomargarita akajimensis	ADE54506.1	0.91	0.25	6
d__Bacteria;p__SAR324;c__SAR324;o__SAR324;f__JACQKK01;g__JACZRV01;s__JACZRV01 sp022574555	MCH8841650.1	0.91	0.67	7
d__Bacteria;p__Methylomirabilota;c__Methylomirabilia;o__Rokubacteriales;f__CSP1-6;g__40CM-4-69-5;s__40CM-4-69-5 sp001917535	OLC36608.1	0.9	0.66	8
d__Bacteria;p__Acidobacteriota;c__Vicinamibacteria;o__Vicinamibacterales;f__UBA823;g__UBA11600;s__UBA11600 sp002717745	MBE41255.1	0.85	0.5	9
d__Bacteria;p__Moduliflexota;c__Moduliflexia;o__Moduliflexales;f__;g__;s__	HGH08997.1	0.91	0.79	10
d__Bacteria;p__Aquificota;c__Aquificae;o__Aquificales;f__Aquificaceae;g__Hydrogenobacter;s__Hydrogenobacter thermophilus	QWK20176.1	0.93	0.79	11
d__Bacteria;p__Tectomicrobia;c__Entotheonellia;o__Entotheonellales;f__Entotheonellaceae;g__Entotheonella;s__Entotheonella palauensis	ETX00812.1	0.91	0.78	12
d__Bacteria;p__Nitrospinota;c__Nitrospinia;o__Nitrospinales;f__Nitrospinaceae;g__Nitromaritima;s__Nitromaritima sp018647025	MBT5259169.1	0.92	0.79	13
d__Bacteria;p__Myxococcota_A;c__UBA9160;o__UBA9160;f__UBA6930;g__GCA-2687015;s__GCA-2687015 sp002687015	MAE95849.1	0.88	0.40	14
d__Bacteria;p__Desulfobacterota_B;c__Binatia;o__Bin18;f__Bin18;g__Bin18;s__Bin18 sp002238415	MCY4387739.1	0.89	0.75	15
d__Bacteria;p__Poribacteria;c__WGA-4E;o__WGA-4E;f__JADFGN01;g__JADFGN01;s__JADFGN01 sp022567695	MCH8292390.1	0.91	0.57	16
d__Bacteria;p__Latescibacterota;c__UBA2968;o__UBA8231;f__UBA8231;g__;s__	NKB70331.1	0.91	0.79	17
d__Bacteria;p__Gemmatimonadota;c__Gemmatimonadetes;o__Longimicrobiales;f__RSA9;g__JACQPS01;s__JACQPS01 sp016207445	MBI4409569.1	0.88	0.68	18
d__Bacteria;p__Gemmatimonadota;c__Gemmatimonadetes;o__Gemmatimonadales;f__GWC2-71-9;g__JADJOM01;s__JADJOM01 sp016794805	MBL8976918.1	0.91	0.56	19
d__Bacteria;p__Gemmatimonadota;c__Gemmatimonadetes;o__Gemmatimonadales;f__Gemmatimonadaceae;g__SCN-70-22;s__SCN-70-22 sp016704465	MBK6489536.1	0.9	0.77	20
d__Bacteria;p__Gemmatimonadota;c__Gemmatimonadetes;o__Gemmatimonadales;f__Gemmatimonadaceae;g__Gemmatirosa;s__Gemmatirosa kalamazoonensis	AHG91797.1	0.95	0.85	21
d__Bacteria;p__Marinisomatota;c__Marinisomatia;o__Marinisomatales;f__TCS55;g__TCS55;s__TCS55 sp012964665	HIB26529.1	0.88	0.63	22
d__Bacteria;p__KSB1;c__UBA2214;o__CR04bin15;f__CR04bin15;g__N075bin58;s__N075bin58 sp004356825	MCH7679494.1	0.92	0.83	23
d__Bacteria;p__Calditrichota;c__Calditrichia;o__Calditrichales;f__Calditrichaceae;g__WJIP01;s__WJIP01 sp014730185	MBD3223402.1	0.92	0.83	24
d__Bacteria;p__Bacteroidota;c__Ignavibacteria;o__Ignavibacteriales;f__Melioribacteraceae;g__JAIPBD01;s__JAIPBD01 sp021739765	MCF8260072.1	0.94	0.8	25
d__Bacteria;p__Bacteroidota;c__Rhodothermia;o__Rhodothermales;f__VXPQ01;g__VXPQ01;s__VXPQ01 sp009839485	MXW63458.1	0.9	0.4	26
d__Bacteria;p__Bacteroidota;c__Rhodothermia;o__Balneolales;f__Balneolaceae;g__SW132;s__SW132 sp009909035	NBC27528.1	0.92	0.75	27

d__Bacteria;p__Bacteroidota;c__Bacteroidia;o__Chitinophagales;f__Saprospiraceae;g__M3007;s__M3007 sp020847425	MCC6282148.1	0.91	0.61	28
d__Bacteria;p__Bacteroidota;c__Bacteroidia;o__Flavobacteriales;f__Flavobacteriaceae;g__MS024-2A;s__MS024-2A sp018699935	MBT6160684.1	0.91	0.82	29
d__Bacteria;p__Pseudomonadota;c__Gammaproteobacteria;o__Nevskiales;f__Salinisphaeraceae;g__Salinisphaera;s__Salinisphaera orenii	ROO28324.1	0.91	0.45	30
d__Bacteria;p__Pseudomonadota;c__Gammaproteobacteria;o__Nevskiales;f__Nevskiaceae;g__Fontimonas;s__Fontimonas thermophila	SFF32314.1	0.92	0.66	31
d__Bacteria;p__Pseudomonadota;c__Gammaproteobacteria;o__Xanthomonadales;f__Rhodanobacteraceae;g__Dokdonella;s__Dokdonella koreensis	ANB19507.1	0.91	0.74	32
d__Bacteria;p__Pseudomonadota;c__Gammaproteobacteria;o__Xanthomonadales;f__Xanthomonadaceae;g__Stenotrophomonas;s__Stenotrophomonas koreensis	KRG60484.1	0.9	0.57	33
d__Bacteria;p__Pseudomonadota;c__Gammaproteobacteria;o__UBA4486;f__UBA4486;g__SMWN01;s__SMWN01 sp003645215	RKZ45139.1	0.91	0.69	34
d__Bacteria;p__Pseudomonadota;c__Gammaproteobacteria;o__Chromatiales;f__Sedimenticolaceae;g__Thiodiazotropha;s__Thiodiazotropha sp003676145	RLW63372.1	0.91	0.73	35
d__Bacteria;p__Pseudomonadota;c__Gammaproteobacteria;o__Enterobacterales_A;f__Alteromonadaceae;g__Aliidiomarina;s__Aliidiomarina haloalkalitolerans	RUO19112.1	0.91	0.76	36
d__Bacteria; p__Pseudomonadota; c__Gammaproteobacteria; o__Enterobacterales; f__Enterobacteriaceae; g__Rahnella; s__Rahnella aceris	QBJ07205.1	0.88	0.72	37
d__Bacteria;p__Pseudomonadota;c__Gammaproteobacteria;o__Pseudomonadales;f__Moraxellaceae;g__Acinetobacter;s__Acinetobacter baumannii	QBM39067.1	0.9	0.72	38
d__Bacteria; p__Pseudomonadota; c__Gammaproteobacteria; o__Oceanospirillales; f__Halomonadaceae; g__Halomonas; s__Halomonas elongata	CBV43776.1	0.92	0.54	39
d__Bacteria;p__Pseudomonadota;c__Gammaproteobacteria;o__Pseudomonadales;f__Pseudomonadaceae;g__Pseudomonas_E;s__Pseudomonas_E putida_L	OLS63570.1	0.93	0.63	40
Bacteria, Gemmatimonadota, Gemmatimonadetes, Gemmatimonadales, Gemmatimonadaceae, Gemmatimonas	MBA3853397.1	0.83	0.61	-
Bacteria, Gemmatimonadota, Gemmatimonadetes, Gemmatimonadales, Gemmatimonadaceae	MBC7672866.1	0.91	0.79	-

Table S4: Primer table. Primers used in this study to perform site-directed mutagenesis (SDM). Reverse primers (designated 'rev') are shown as reverse complement sequences.

Name	Sequence	Purpose
yncD_Q84A_fw	GAACTATGCGgcgGATTTACAGCTGTCGATTC	SDM of pBAD-yncD for point mutation of Q84A.
yncD_Q84A_rev	TGCCGGTTTTGTACCTGC	SDM of pBAD-yncD for point mutation of Q84A.
yncD_Y99A_fw	CCGCTCCACTgcgGGTATTCGCGG	SDM of pBAD-yncD for point mutation of Y99A.
yncD_Y99A_rev	GAGCCAAATCCGCGAATC	SDM of pBAD-yncD for point mutation of Y99A.
yncD_K274E_fw	CGACACGCGAgagACCATCAAGC	SDM of pBAD-yncD for point mutation of K274E.
yncD_K274E_rev	TACTGTTCTGCACGAGGC	SDM of pBAD-yncD for point mutation of K274E.
yncD_R304E_fw	TGCCGGAGAGgagGAAACGACCC	SDM of pBAD-yncD for point mutation of R304E.
yncD_R304E_rev	TACATCATCACACTCATATC	SDM of pBAD-yncD for point mutation of R304E.
yncD_Q308A_fw	AGAAACGACCgcgTACCAGTCAATACCC	SDM of pBAD-yncD for point mutation of Q308A.
yncD_Q308A_rev	CGCTCTCCGGCATACATC	SDM of pBAD-yncD for point mutation of Q308A.
yncD_Q310A_fw	GACCCAGTACgcgTCAATACCCATGGC	SDM of pBAD-yncD for point mutation of Q310A.
yncD_Q310A_rev	GTTTCTCGCTCTCCGGCA	SDM of pBAD-yncD for point mutation of Q310A.
yncD_R365E_fw	GAGTGAAAACgagAAGGGCTACAATAAC	SDM of pBAD-yncD for point mutation of R365E.
yncD_R365E_rev	ATGTTTTCGTAGTTCAGG	SDM of pBAD-yncD for point mutation of R365E.
yncD_N477A_fw	GCCGACGATTgcgGAGCTGTCTTATC	SDM of pBAD-yncD for point mutation of N477E.
yncD_N477A_rev	GTTTCAAAACCTCGCCCG	SDM of pBAD-yncD for point mutation of N477E.
yncD_Y481A_fw	TGAGCTGTCTgcgCGTGCTGATG	SDM of pBAD-yncD for point mutation of Y481A.
yncD_Y481A_rev	TTAATCGTCGGCGTTTCA	SDM of pBAD-yncD for point mutation of Y481A.
yncD_R536E_fw	TAGCGGTGGGgagACGACTTACAAAAATG	SDM of pBAD-yncD for point mutation of R536E.
yncD_R536E_rev	CTGCTATCGACAACAATTTC	SDM of pBAD-yncD for point mutation of R536E.

Table S5: Plasmid table. Plasmids used in this study.

Name	Purpose
p20b-yncD	IPTG-inducible plasmid for the overexpression of pqqU (yncD) in *Escherichia coli* C41(DE3).
pBAD-yncD	Rhamnose-inducible plasmid for pqqU complementation cultures of *Escherichia coli* ΔyncDΔpts.
pBAD-His10-yncD	Rhamnose-inducible plasmid for expression of N-terminallt-tagged pqqU for immunoblotting to validate presence of PqqU.
pBAD-His10-yncD-K274E	Rhamnose-inducible plasmid for expression of N-terminallt-tagged pqqU for immunoblotting to validate presence of PqqU with K274E mutation.
pBAD-His10-yncD-R304E	Rhamnose-inducible plasmid for expression of N-terminallt-tagged pqqU for immunoblotting to validate presence of PqqU with R304E mutation.
pBAD-His10-yncD-R365E	Rhamnose-inducible plasmid for expression of N-terminallt-tagged pqqU for immunoblotting to validate presence of PqqU with R365E mutation.

Table S6: Strain table. Bacterial strains used in this study.

Name	Genotype	Purpose
Escherichia coli DH5α	F– ϕ80lacZΔM15 Δ(lacZYA-argF)U169 recA1 endA1 hsdR17(rK–, mK+) phoA supE44 λ–thi-1 gyrA96 relA1	Cloning strain for site-directed mutagenesis of pBAD-yncD plasmid.
Escherichia coli C41(DE3)	F – ompT hsdSB (rB- mB-) gal dcm (DE3)	Expression strain for His-tagged pqqU (yncD).
Escherichia coli ΔptsΔpqqU	F- λ- rph-1 Δ(araD-araB)567 Δ(lacZ4787(::rrnB-3) Δ(rhaD-rhaB)568 hsdR514 yncD::scar Δ(ptsHIcrr)::kan	PqqU complementation cultures with plasmid pBAD-yncD. Incapable of metabolizing glucose via the EMP glycolysis pathway.

SUPPLEMENTAL MOVIES

Movie S1: Contouring of coulomb potential map for the PqqU-PQQ structure

Movie S2: Morph of conformational changes in loops 7 and 8 between apo and PQQ-bound PqqU

Movie S3: Morph of conformational changes in the PqqU binding pocket sidechains between apo and PQQ-bound PqqU

SUPPLEMENTAL DATA

Supplemental data S1: AF2 models of the PqqU-phage proteins complex

Supplemental data S2: Full output sheet of PqqU homologs, associated metadata and quinoproteins identified

Supplemental data S3: Multiple alignment file of PqqU homologs

Supplemental data S4: Chai-1 structural models of diverse PqqU homologs in complex with PQQ
